# A Logical Study of Moral Responsibility

**DOI:** 10.1007/s10670-023-00730-2

**Published:** 2023-09-04

**Authors:** Hein Duijf

**Affiliations:** Fakultät für Philosophie,Wissenschaftstheorie und Religionswissenschaft Munich Center for Mathematical Philosophy, Ludwig-Maximilians-Universität München, Geschwister-Scholl-Platz 1, 80539 Munich, Germany

## Abstract

This paper proposes a logical framework for studying the structure of moral responsibility for outcomes. The analysis incorporates two vital features: an agency condition and a negative condition of an alternative possibility. The logical language allows us to identify and disambiguate seven plausible criteria for moral responsibility. To accommodate interdependent decision contexts, the semantics are given in terms of so-called responsibility games. The logical framework enables us to classify the logical relations between these seven criteria for moral responsibility. Although all seven criteria are logically distinct, I also identify circumstances where the seven criteria locally reduce to only three.

## Introduction

What are the general conditions when a person can be ascribed moral responsibility for a given outcome? A theory of moral responsibility needs to determine who is to be held morally responsible and for what. Such theories are needed to correctly allocate moral responsibility in complex decision processes that involve several stages and multiple decision-makers. Although philosophers disagree on the exact conditions, by representing moral responsibility mathematically, we can enhance our (conceptual and practical) capacity to determine which decision-makers are morally responsible for a particular outcome.

My aim is to introduce a logical framework for studying the structure of moral responsibility for outcomes.^1^ My analysis is based on the commonly recognized intuition that agents can be held morally (co-)responsible for a given outcome if and only if they are somehow involved in the process that brought about the morally undesirable outcome and their involvement was avoidable. In other words, moral responsibility for outcomes requires the positive *agency condition* and the *negative condition* of an alternative possibility.^2^

The relationship between an agent’s choices and the morally relevant state of affairs take center stage in this paper. This relationship can be conceptualized in multiple ways. I focus on two basic conceptions. First, whenever an agent does not causally contribute to a given outcome, then she cannot be held responsible for its realization. Or, equivalently, an agent can be held responsible for the realization of a given outcome only if she *causally contributes* to its realization. This means that judgements about the causal contributions are indispensable for a theory of moral responsibility.

At this stage, the second conception is stated most clearly in terms of probabilities, where probabilities can be taken to represent an agent’s justified degrees of beliefs: an agent can be held responsible for the realization of a given outcome only if she *raises the probability* of its realization. In other words, only if there is no alternative action in the agent’s repertoire whose performance would increase the probability of realizing that outcome. It is important to emphasize that I will also give an interpretation of this intuition in situations where probabilities are unavailable or nonsensical.

My logical framework for moral responsibility consists of a logical language and semantics, where the logical language is interpreted using certain models (see below). In the first part, I will introduce a *logical language* that can express the aforementioned two basic conceptions. One of the benefits of using a logical language is that it becomes possible to identify and distinguish alternative criteria for moral responsibility on the basis of these two initial ideas. Each of the two basic conceptions is formalized by an associated logical operator: the statement that agent *i* causally contributes to realizing the state of affairs $\varphi$ is formalized by $[i\;\textsf{caus}]\varphi$; the statement that agent *i* promotes the realization of the state of affairs $\varphi$ is formalized by $[i\;\textsf{prom}]\varphi$ (i.e. the agent raises the probability of $\varphi$). Given these logical operators and standard boolean connectives one can directly formulate *alternative criteria for moral responsibility*: for example, $\lnot [i\;\textsf{prom}]\lnot \varphi$ characterizes that it is not the case that agent *i* raises the probability that $\varphi$ does not obtain; and $[i\;\textsf{prom}][i\;\textsf{caus}]\varphi$ characterizes that agent *i* raises the probability that she herself causally contributes to the realization of $\varphi$. My study will be confined to seven criteria for moral responsibility: three criteria have a simple logical form and four have a complex logical form (see Sect. 2, for more details). Although many formalisms seem to only rely on the simple criteria, the fruitfulness of the complex criteria can be illustrated by locating the anatomy of moral responsibility by Braham and van Hees (2012) within my logical formalism: their theory states that agent *i* is morally responsible for $\varphi$ if and only if the conjunction of $[i\;\textsf{caus}]\varphi$ and $\lnot [i\;\textsf{prom}]\lnot [i\;\textsf{caus}]\varphi$ obtains.

In the second part, I will provide truth-conditions for these logical operators in terms of models that I call *responsibility games*. First, I will discuss some options for interpreting the statement that an agent causally contributes to the realization of a given state of affairs. It is not my aim to come up with the best way of modelling this statement, rather, it is my aim to cast a certain way of modelling causal contributions within a logical formalism. In particular, I will use logics of agency within the tradition of *seeing to it that* (Belnap et al., 2001; Horty, 2001) and the idea of the but-for test and the NESS test (Hart and Honoré, 1959; Honoré, 1997; Wright, 1988; 2011) to cash out some important considerations in modelling causal contributions.^3^ Second, I will provide an interpretation for the statement that an agent (weakly) raises the probability that a given state of affairs is realized. Since this way of spelling out the second factor requires probabilities, I propose to view this as a theory of moral responsibility under risk.^4^

Third, one of my aims is to also propose a framework that covers decision-making under *uncertainty*, as opposed to *risk*. I will present an interpretation for the fact that an agent promotes the realization of a given state of affairs in cases where probabilities are absent. After all, there are cases where probabilities are unknown or not meaningful. I propose that these yield two interpretations for saying that a given agent promotes the realization of a given state of affairs, depending on whether the decision context is to be viewed as involving risk or as involving uncertainty.^5^

Given these seven criteria for moral responsibility, one may ask whether they are truly logically distinct. One way to argue that certain criteria are logically distinct is by crafting (everyday) counterexamples. However, such examples cannot be used to argue for certain logical implications and validities. To determine such validities and invalidities in a systematic way, we need a formal theory of the criteria for moral responsibility. The logical machinery provides the opportunity to explore the logical (in)dependencies between the seven criteria for moral responsibility. In the third part, I will explore the *logical classification* of these criteria for moral responsibility. Roughly stated, all seven criteria for moral responsibility are logically distinct, while some criteria for moral responsibility are logically broader than others and others are logically unrelated (see Sect. 6 and Fig. 3 for more details).

One may ask under which conditions certain criteria for moral responsibility are in full agreement. The logical framework can be used to identify circumstances where certain criteria agree. That is, in certain circumstances, the seven criteria for moral responsibility can be *locally reduced* to just three. I will identify three circumstances where this local reduction is possible. The first consists of scenarios of decision-making under uncertainty (as opposed to risk). One of the results is that the four complex criteria for moral responsibility are only viable in scenarios of decision-making under risk (i.e., where probabilities are available). The other two circumstances are subclasses of scenarios of decision-making under risk (see Sect. 8 and Fig. 7 for more details). In these circumstances, the four complex criteria for moral responsibility can, once again, be reduced to the three simpler criteria. Hence, for a wide class of cases we can make do with the simple criteria for moral responsibility.

The paper is organized as follows. The logical language is introduced in Sect. 2 and used to identify seven criteria for moral responsibility. In Sect. 3, I introduce responsibility games. In Sect. 4, I discuss the truth-conditions of statements of the form ‘agent *i* causally contributes to the realization of $\varphi$’. In Sects. 5 and 7 I present the truth-conditions for statements of the form ‘agent *i* promotes the realization of $\varphi$’ for decision-making under risk and under uncertainty, respectively. Finally, I provide a logical classification of the seven criteria for moral responsibility in Sect. 6 and I prove that the four complex criteria for moral responsibility can be locally reduced to three simple criteria in three natural circumstances in Sect. 8. A discussion concludes the paper. All results are proved in the appendix.

## The Logical Characterization of Moral Responsibility

This paper focuses on moral responsibility for outcomes. More precisely, the aim is to provide a characterization for the statement that a given agent is morally responsible for the realization of a given state of affairs. Before proceeding, I should clarify that theories of moral responsibility typically include an autonomy condition of the form:

Autonomy Condition: The person is an autonomous agent who performed his or her action intentionally (Braham and van Hees, 2012, p. 605)^6^

The kind of autonomy and intentionality that is required for moral responsibility will not be analysed here. Rather, I will simply assume that this condition is satisfied. This allows me to focus on my main contribution: the development of a logical framework for studying the structure of moral responsibility, especially the positive agency condition and the negative condition of an alternative possibility.

Let me continue with the logical framework. To begin, the formal language includes a constant $0$ which denotes a particular *morally undesirable* state of affairs.^7^ The formal language also includes the standard classical connectives, $\lnot$, $\wedge$, $\vee$, and $\rightarrow$. I will use $1$ as an abbreviation for $\lnot 0$, that is, $1$ denotes the fact that the morally undesirable state of affairs is avoided.^8^

The logical constant $0$ is not analysed in further detail here. Depending on one’s ethical inclinations, one may consider different morally undesirable states of affairs. The framework is neutral on this issue and assumes that a particular morally undesirable state of affairs has been identified.^9^ One important take home message of the present paper is that there is scope for ambiguity and/or disagreement regarding the criteria for moral responsibility *even if* there is clarity and agreement on which state of affairs is morally undesirable.

As indicated in the introduction, my logical characterization of moral responsibility essentially relies on two ideas:

- *Causal contributions.* The statement that a given agent *i* causally contributes to the realization of a given state of affairs $\varphi$ is formalized by $[i\;\textsf{caus}]\varphi$.
- *Promotion of states of affairs.* The statement that a given agent *i* promotes the realization of $\varphi$ is formalized by $[i\;\textsf{prom}]\varphi$. For example, in decision-making under risk, this statement roughly means that agent *i* promotes the probability that $\varphi$ is realized, that is, she (weakly) raises the probability that $\varphi$ is realized. In other words, it says that there is no alternative action in agent *i*’s repertoire whose performance would increase the probability of achieving $\varphi$.

Moreover, to characterize the negative condition, the language needs the capacity to express alternative possibilities. As is standard in modal logic, I will use $\Diamond \varphi$ to formalize statements of the form ‘it is possible that $\varphi$ holds’.

Finally, the formal language ${\mathfrak {L}}$ under consideration in this paper assumes a given set *N* of agents and is the smallest set (in terms of set-theoretic inclusion) that satisfies the following conditions:

1. $$0\in {\mathfrak {L}}$$; (Constant $$0$$)
2. if $\varphi \in {\mathfrak {L}}$, then $\lnot \varphi \in {\mathfrak {L}}$; (Negation)
3. if $\varphi ,\psi \in {\mathfrak {L}}$, then $\varphi \wedge \psi \in {\mathfrak {L}}$; (Conjunction)
4. if $\varphi \in {\mathfrak {L}}$ and $i\in N$, then $[i\;\textsf{caus}]\varphi \in {\mathfrak {L}}$; (Causal Contribution)
5. if $\varphi \in {\mathfrak {L}}$ and $i\in N$, then $[i\;\textsf{prom}]\varphi \in {\mathfrak {L}}$; (Promotion)
6. if $\varphi \in {\mathfrak {L}}$, then $\Diamond \varphi \in {\mathfrak {L}}$.(Possibility)

I omit parentheses, brackets, and braces if the omission does not give rise to ambiguities. The operators $\vee$, $\rightarrow$, and $\leftrightarrow$ abbreviate the classical propositional constructs.

I would like to demonstrate that this logical language is able to express different criteria for moral responsibility. The aim of this paper is not to argue for the plausibility of these criteria. Rather, the aim is to explicate these criteria and investigate their logical connections. On a more constructive view, the following criteria can be thought of as primitives for modelling moral responsibility; the adequate notion of moral responsibility can be specified as a combination of some of these primitives. One possibility is to postulate moral responsibility in terms of a list of necessary and sufficient conditions.

The first criterion for moral responsibility can be represented by:

1 $$\begin{aligned}{}[i\;\textsf{caus}]0, \end{aligned}$$

which characterizes that agent *i* causally contributes to the realization of $0$. The idea is that an agent cannot be held responsible for a given state of affairs if she did not causally contribute to its realization. Or, equivalently, if an agent can be held responsible for a given state of affairs, then she causally contributed to its realization. Although it is standard to assume that causal responsibility alone is not sufficient for moral responsibility, many theories of moral responsibility take causal responsibility to be one of its necessary elements.^10^

The second criterion is represented by

2 $$\begin{aligned}{}[i\;\textsf{prom}]0, \end{aligned}$$

which characterizes that agent *i* promotes the realization of $0$. The idea is that an agent cannot be held responsible for a given state of affairs if she did not promote its realization. In other words, an agent is responsible for the realization of a given state of affairs only if there is no alternative action in the agent’s repertoire whose performance would increase the probability of achieving that state of affairs. Or, equivalently, only if the agent is performing an action that has the maximum probability of achieving that state of affairs.

In addition, the logical language can be utilized to investigate plausible alternative criteria for moral responsibility. Consider the following formula:

3 $$\begin{aligned} \lnot [i\;\textsf{prom}]\lnot 0\quad (\leftrightarrow \lnot [i\;\textsf{prom}]1), \end{aligned}$$

which characterizes that it is not the case that agent *i* promotes the realization of $1$. Or, equivalently, it is not the case that agent *i* promotes the avoidance of $0$. The idea is that an agent can be held responsible for a given state of affairs only if there was an alternative action in the agent’s repertoire whose performance would decrease the probability of achieving that state of affairs. Or, equivalently, only if the agent is performing an action that does not have the minimum probability of achieving that state of affairs.^11^

In contrast to the above criteria for moral responsibility, Braham and van Hees (2012, 2018) argue that you can escape moral responsibility if you choose “a strategy that reduces the probability that your action [made a causal contribution to] an outcome; it is not about you having full control over that outcome” (Braham and van Hees, 2018, p. F99).^12^ This condition is represented by the following formula:

4 $$\begin{aligned} \lnot [i\;\textsf{prom}]\lnot [i\;\textsf{caus}]0, \end{aligned}$$

which represents the fact that it is not the case that agent *i* promotes that she does not causally contribute to the realization of $0$. Accordingly, an agent is responsible for the realization of $0$ only if the agent is performing an action that does not have the maximum probability of achieving $\lnot [i\;\textsf{caus}]0$. Hence, only if the agent is performing an action that does not have the minimum probability that she causally contributes to the realization of $0$. The idea is that an agent cannot be held responsible for a given state of affairs if she promoted that she herself did not causally contribute to its realization. That is, she escapes moral criticism if she promotes that the causal link between herself and the state affairs is severed.

One may wonder whether these are all the available criteria for moral responsibility. A *moral responsibility operator* could be defined as any sequence of zero or more negations, causal-contribution operators, and promotion-operators appended to the constant $0$. Such an operator can be defined as positive or negative according to whether it contains an odd or even number of negations. The criteria for moral responsibility presented above are negative moral responsibility operators.^13^ It is plausible to assume that these negative moral responsibility operators concern *blameworthiness* and that *praiseworthiness* relates to the positive moral responsibility operators. It is therefore best to view the current analysis as being restricted to blameworthiness.

In the present paper, I restrict my investigation to the seven negative moral responsibility operators with the simplest logical form. From the perspective of their logical form, criteria (1)–(3) are *simple* moral responsibility operators whereas criterion (4) is *complex*. The logical framework yields three alternative complex criteria of moral responsibility, bringing the total to seven. Briefly stated, the remaining three are:

(5) $$[i\;\textsf{prom}][i\;\textsf{caus}]0$$;
  An agent can only be held morally responsible for a given state of affairs if she promotes that she herself causally contributes to its realization.
(6) $$\lnot [i\;\textsf{prom}][i\;\textsf{caus}]1$$;
  An agent can only be held morally responsible for a given state of affairs if it is not the case that she promotes that she causally contributes to avoiding its realization.
(7) $$[i\;\textsf{prom}]\lnot [i\;\textsf{caus}]1$$;
  An agent can only be held morally responsible for a given state of affairs if she promotes that it is not the case that she causally contributes to avoiding its realization.

At this stage, one of the key benefits of formalization should be clear: The logical language helps to disambiguate various criteria for moral responsibility in a meticulous way. Doing so in natural language is not only a hassle, but it is open to the risk of conflating some of these criteria. Moreover, the logical representations of the four complex criteria for moral responsibility demonstrates that the two initial ideas of this paper can be combined in various ways.

Before closing this section, it should be noted that the aforementioned criteria could be viewed as possible formalizations of the agency condition. But, as indicated in the introduction, many philosophers think that moral responsibility also involves a negative condition of an alternative possibility. Each of the aforementioned formalizations of the agency condition comes with its associated formalization of this negative condition: for each agency condition formalized by a formula $\chi$, the corresponding negative condition is formalized by $\Diamond \lnot \chi$. After all, $\Diamond \lnot \chi$ expresses that it is possible that the agent does not satisfy the agency condition $\chi$. For example, for the agency condition (2) $[i\;\textsf{prom}]0$, the corresponding negative condition is $\Diamond \lnot [i\;\textsf{prom}]0$, which expresses that it is possible that agent *i* does not promote the realization of $0$. Hence, we get the following negative conditions:

1. $\Diamond \lnot [i\;\textsf{caus}]0$, which states that it is possible that agent *i* does not causally contribute to the realization of $0$.
2. $\Diamond \lnot [i\;\textsf{prom}]0$, which states that it is possible that agent *i* does not promote the realization of $0$.
3. $\Diamond [i\;\textsf{prom}]1$, which states that it is possible that agent *i* does promote the realization of $1$.
4. $\Diamond [i\;\textsf{prom}]\lnot [i\;\textsf{caus}]0$, which states that it is possible that agent *i* promotes that she does not causally contribute to the realization of $0$.
5. $$\Diamond \lnot [i\;\textsf{prom}][i\;\textsf{caus}]0$$.
6. $$\Diamond [i\;\textsf{prom}][i\;\textsf{caus}]1$$.
7. $$\Diamond \lnot [i\;\textsf{prom}]\lnot [i\;\textsf{caus}]1$$.

From a logical perspective, it is of interest to investigate whether these seven criteria are logically distinct. Moreover, it is important to know under which circumstances these seven criteria for moral responsibility come apart and when they are identical. The answers to these questions inevitably depend on the exact interpretation of these logical operators. In the remainder of the paper I provide semantics for these logical operators (Sects. 3, 4, 5, and 7), and will indicate that the answers to some the above questions depend on whether one is considering decision-making under risk or under uncertainty (Sects. 6 and 8).

## Responsibility Games

I propose to use responsibility games, which are an adaptation and extension of the models of game theory, to interpret the logical language. In this section, I will introduce these responsibility games and in the next sections I will provide truth conditions for the logical language. A *game form* represents an interdependent decision context involving a finite set *N* of individual agents at a given moment in time. Each individual agent *i* is assigned a finite set of available actions $A^{}_{i}$. The Cartesian product $\times _{i\in N}A^{}_{i}$ of all individual agents’ sets of available actions gives the full set *A* of action profiles.

### **Definition 1**

*(Game Form)* A *game form* *S* is a tuple $\langle N,(A^{}_i)\rangle$, where *N* is a finite set of individual agents, for each agent *i* in *N* it holds that $A^{}_i$ is a non-empty and finite set of actions available to agent *i*. The set of action profiles *A* is given by $\times ^{}_{i\in N}A^{}_i$.

Let me mention some notational conventions: For each group ${\mathcal {G}}\subseteq N$, the set $A^{}_{{\mathcal {G}}}$ of group actions available to ${\mathcal {G}}$ is defined as the Cartesian product $\times _{i\in {\mathcal {G}}} A^{}_{i}$. I use $a^{}_{{\mathcal {G}}}, b^{}_{{\mathcal {G}}}, a'_{{\mathcal {G}}}$ as variables for elements of $A^{}_{{\mathcal {G}}}$ and I let $-{\mathcal {G}}$ denote the relative complement $N\setminus {\mathcal {G}}$. Finally, any group actions $a^{}_{{\mathcal {G}}}\in A^{}_{{\mathcal {G}}}$ and $b^{}_{-{\mathcal {G}}}\in A^{}_{-{\mathcal {G}}}$ can be combined into an action profile $(a^{}_{{\mathcal {G}}}, b^{}_{-{\mathcal {G}}})\in A$.

For my present study, I focus primarily on the realization of states of affairs. Because I assume that an action profile fully determines the future state of the world, a state of affairs can be identified with a set of action profiles. Intuitively, a state of affairs $X\subseteq A$ is represented by those aspects that all outcomes of the action profiles in *X* have in common. In a similar way, it is standard in logic to think of a proposition as a set of possible worlds.

A *responsibility game* can be taken to represent an interdependent decision context and can be used to study responsibility with respect to a given morally undesirable state of affairs.^14^ For example, when a committee uses a voting procedure to decide whom to hire and ended up hiring a bad candidate, one might be interested in studying the responsibility with respect to the appointment of the candidate. In such a scenario, the voting procedure can be represented by a game form, and the appointment of the bad candidate can be represented by a state of affairs.

### **Definition 2**

*(Responsibility Game)* A *responsibility game* *S* is a tuple $\langle N, (A^{}_{i}), V\rangle$, involving a game form $\langle N,(A^{}_i)\rangle$ and a morally undesirable state of affairs $V\subseteq A$. To picture the morally undesirable state of affairs $V$, I often use a utility function *u* where

$$\begin{aligned}u(a)= {\left\{ \begin{array}{ll} 0,\,{ if}a\in V\\ 1,\,{ otherwise}. \end{array}\right. }\end{aligned}$$

Given such a responsibility game, we use $I:=A\setminus V$ to denote the morally desirable state of affairs.

It is vital to emphasize the conceptual disparity between responsibility games and the models of game theory, as they are used in rational choice theory (despite the formal similarities). For example, even though both rely on utilities, the interpretation of these utilities differs: in responsibility games the binary utilities represent some morally relevant state of affairs, whereas in game theory it is standard to use utilities to represent an agent’s choice behaviour or revealed preferences.^15^

**Fig. 1:**
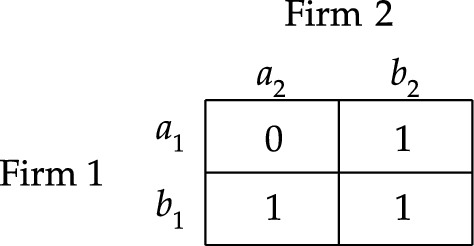
Responsibility game $$S_{1}$$

Let me illustrate these responsibility games with an example. Consider an example where two firms bring about a harm by simultaneously emitting amounts of some toxin into a river. Moreover, no firm could have brought about the harm alone. We can simplify the scenario by assuming that each firm *i* has two available options: to emit the toxin into the river or to refrain from doing so, represented by $a^{}_{i}$ and $b^{}_{i}$ respectively. Then, this example can modelled by action profile $a=(a^{}_{1}, a^{}_{2})$ in responsibility game $S_{1}$ depicted in Fig. 1. After all, the morally undesirable outcome will result if and only if the firms both emit their toxins into the river.

Let me end with stating the evaluation rules for the logical constant $0$ and the boolean connectives:

### **Definition 3**

*(Evaluation Rule: Propositional Connectives)* Let $S=\langle N, (A^{}_{i}), V\rangle$ be a responsibility game, let $a\in A$ be an action profile, and let $\varphi$ be a formula. Then, we say that $\varphi$ holds at *a* in *S*, notation: $S,a\models \varphi$, if and only if:

$$\begin{aligned} \begin{array}{lll} S,a\models 0&{} \text{iff} &{} a\in V.\\ S,a\models \lnot \varphi &{} \text{iff} &{} S,a\not \models \varphi .\\ S,a\models \varphi \wedge \psi &{} \text{iff} &{} S,a\models \varphi \text{ and } S,a\models \psi .\\ S,a\models \varphi \vee \psi &{} \text{iff} &{} S,a\models \varphi \text{ or } S,a\models \psi .\\ \end{array} \end{aligned}$$

As is standard in the logical tradition, we say that a given formula holds in *S*, notation $S\models \varphi$, if and only if every action profile $a$ in *﻿S* satisfies $S,a\models \varphi$. Moreover, we say that a given formula is valid, notation: $\models \varphi$, if and only if every responsibility game *S* satisfies $S\models \varphi$.

## Causal Contributions: NESS Conditions

The aim of this section is to provide truth conditions for statements of the form ‘agent *i* causally contributes to the realization of $\varphi$’. As a precursor to studying causal contributions, it is important to check whether the actions of a given group of agents are sufficient in order to guarantee a certain state of affairs. To do so, I follow the theory of ‘seeing to it that’, often abbreviated to *stit theory*, which relies on a modal treatment of agency (Belnap et al., 2001; Horty, 2001).^16^ The central idea in stit theory is to characterize the statement that *group* ${\mathcal {G}}$ *sees to it that a given state of affairs* $\varphi$ *is realized* by a modal operator of the form $[{\mathcal {G}}\;\textsf{stit}]\varphi$. In other words, this operator characterizes the fact that group ${\mathcal {G}}$ guarantees the realization of $\varphi$, regardless of the actions of the others.^17^

To express the negative condition of an alternative possibility I propose to characterize statements of the form *it is possible that* $\varphi$ by a modal operator of the form $\Diamond \varphi$. In particular, $\Diamond \lnot \varphi$ expresses the fact that it was possible to avoid $\varphi$.

Let me present the truth conditions for these two logical operators:

### **Definition 4**

*(Evaluation Rule: Agency and Alethic Possiblity)* Let *S* be a responsibility game, let $a\in A$ be an action profile, let ${\mathcal {G}}\subseteq N$ be a group of agents, and let $\varphi$ be a formula. Then, the truth-conditions for $[{\mathcal {G}}\;\textsf{stit}]\varphi$ and $\Diamond \varphi$ are given by:

$$\begin{aligned} \begin{array}{lll} S,a\models [{\mathcal {G}}\;\textsf{stit}]\varphi &{} {iff} &{} \text {every}\,\, b\in A \,\,\text {with}\,\, b_{{\mathcal {G}}}=a_{{\mathcal {G}}} \,\, \text {satisfies}\, S,a\models \varphi .\\ S,a\models \Diamond \varphi &{} {iff} &{} \text {there is a profile}\, , b\in A \,\, \text {such that}\,\, S,b\models \varphi . \\ \end{array} \end{aligned}$$

^18^

In other words, we say that $a_{{\mathcal {G}}}$ sees to it that $\varphi$ if and only if for any $c^{}_{-{\mathcal {G}}}\in A^{}_{-{\mathcal {G}}}$ it holds that $\varphi$ is true at $(a^{}_{{\mathcal {G}}}, c^{}_{-{\mathcal {G}}})$.^19^ Hence, a group of agents guarantees the realization of a given state of affairs if and only if that state of affairs obtains regardless of what the group’s non-members do.

As is standard in normal modal logic, each operator gives rise to a dual operator. In particular, the modal operator $\Box \varphi$ abbreviates $\lnot \Diamond \lnot \varphi$ and represents alethic necessity. Note that $\Box \varphi$ is true at a given action profile *a* if and only if $\varphi$ is true at every action profile in the responsibility game. Moreover, the modal operator $\langle {\mathcal {G}}\;\textsf{stit}\rangle \varphi$ abbreviates $\lnot [{\mathcal {G}}\;\textsf{stit}]\lnot \varphi$. It is easy to verify that $\langle {\mathcal {G}}\;\textsf{stit}\rangle \varphi$ is true at a given action profile *a* if and only if there exists a $b\in A$ with $b^{}_{{\mathcal {G}}}=a^{}_{{\mathcal {G}}}$ that satisfies $\varphi$. In other words, $\langle {\mathcal {G}}\;\textsf{stit}\rangle \varphi$ can be taken to mean that group ${\mathcal {G}}$ performs an action that is compatible with the realization of $\varphi$. Or equivalently, that group ${\mathcal {G}}$ performs an action that does not rule out $\varphi$.

It may be tempting to think that an agent causally contributes to the realization of a given state of affairs if and only if her actions guarantee its realization.^20^ Accordingly, criterion (1) for moral responsibility for the realization of $V$ would then be characterized by $[i\;\textsf{stit}]0$. This would mean that moral responsibility requires that the agent’s actions were sufficient to bring about that state of affairs. There are some problems with this intuition that are helpful to briefly discuss. First, there is a common intuition that an agent’s actions do not causally contribute to the realization of a given state of affairs if the agent’s actions have no effect on whether or not that state of affairs is realized. That is, if the state of affairs were unavoidable, then an agent cannot causally contribute to its realization. We can use the logical operator of the form $\Diamond \varphi$ to formalize the statement that it is possible that $\varphi$ obtains. Then, the first problem can be addressed by using the *deliberative stit* operator of the form $[i\;\textsf{dstit}]\varphi$ (Horty and Belnap, 1995; Belnap et al., 2001), where $[i\;\textsf{dstit}]\varphi \leftrightarrow [i\;\textsf{stit}]\varphi \wedge \Diamond \lnot \varphi$.

Notice that this intuition entails that criterion (1) for moral responsibility should be *narrower*. After all, it is easy to see that $\models [i\;\textsf{dstit}]\varphi \rightarrow [i\;\textsf{stit}]\varphi$, which means that deliberately seeing to it that $\varphi$ logically entails guaranteeing $\varphi$. In other words, if an agent is morally responsible under the deliberative stit, then that agent is morally responsible under the regular stit.

Let us now consider the second worry. Reconsider the example of the two firms emitting toxins into the river (see Fig. 1). Notice that [Firm $2\;\textsf{dstit}]0$ is false at *a*. Hence, if we endorse the notion of causality that is characterized by [Firm $2\;\textsf{dstit}]0$, then we would conclude that neither firm causally contributes to the harm they together brought about. This goes against the widely held view in the literature on causality.

One way to address this second worry is to rely on the idea that the agent’s action had to be vital in the realization of $\varphi$. In legal theory, it is common to use the so-called *but-for test* for causation. The idea is that an agent causally contributes to the realization of a given state of affairs if and only if her actions were necessary for its realization. In our logical formalism, this idea can be approximated by the statement $[i\;\textsf{butfor}]\varphi \equiv \varphi \wedge \lnot [N-i\;\textsf{stit}]\varphi$,^21^ which says that $\varphi$ is realized and agent *i* could unilaterally deviate so that $\varphi$ is not realized. Reconsider the example modelled in responsibility game $S_1$ of Fig. 1. Notice that, at action profile *a*, each firm is a but-for condition for the realization of $V$; for example, the statement [Firm $2\;\textsf{butfor}]0$ is true at *a*. The but-for test entails that criterion (1) of moral responsibility should be *broadened*: after all, there are cases where an agent’s actions are a but-for condition for a given state of affairs even though her actions do not guarantee its realization.

**Fig. 2:**
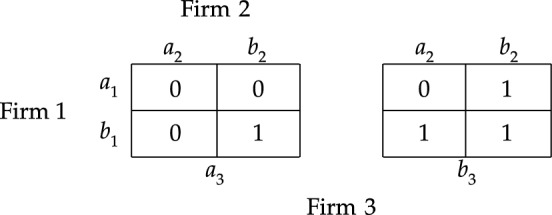
Responsbility game $$S_2$$

However, there are simple examples where the deliberative stit and the but-for test both fail to deliver the intuitively correct answer. To illustrate this, consider the example where three firms bring about a harm by simultaneously emitting amounts of some toxin into a river, in which no firm could have brought about the harm alone, and in which every pair of firms brought about the harm together. We can simplify the scenario by assuming that each firm *i* has two options available: to emit the toxin into the river or to refrain from doing so, represented by $a^{}_{i}$ and $b^{}_{i}$. Then, this scenario can be modelled in terms of responsibility game $S_{2}$ depicted in Fig. 2, and, in particular, by considering action profile $a=(a^{}_{1}, a^{}_{2}, a^{}_{3})$. Notice that both [Firm $2\;\textsf{dstit}]0$ and [Firm $2\;\textsf{butfor}]0$ are false at *a*. Hence, if one endorses the notion of causality that is characterized by the deliberative stit or the but-for test, then one must conclude that none of the firms causally contributes to the harm they together brought about.

The *NESS condition* generalizes the above considerations (Hart and Honoré, 1959; Honoré, 1997; Wright, 1988).^22^ A given condition *C* is a weak NESS condition for the realization of a state of affairs *X* if and only if it is a Necessary Element of a Sufficient Set of conditions. From the perspective of responsibility games, this idea can be spelled out as follows:^23^

### **Definition 5**

*(Evaluation Rule: Causal Contribution)* Let *S* be a responsibility game, let $a\in A$ be an action profile, let $i\in N$ be an agent, and let $\varphi$ be a formula. Then, we say that agent *i* is* a weak NESS condition for* $\varphi$ *at* *a* if and only if there is a group of agents ${\mathcal {G}}\subseteq N$ (possibly a singleton) such that

1. $$S,a\models [{\mathcal {G}}\;\textsf{stit}]\varphi$$, and
2. $$S,a\models \lnot [{\mathcal {G}}-i\;\textsf{stit}]\varphi$$.

In addition, the truth-condition for $[i\;\textsf{caus}]\varphi$ is given by:

$$\begin{aligned} \begin{array}{lll} S,a\models [i\;\textsf{caus}]\varphi &{} {iff} &{} \,\,\text {agent}\,\, i \,\,\text {is a weak NESS condition for}\,\, \varphi \,\,\text {at action profile}\,\, a.\\ \end{array} \end{aligned}$$

^24^

In logics of agency in the tradition of seeing to it that, causality is not often studied directly (exceptions include Xu, 1997; Lorini et al., 2014; Baltag et al., 2021; Sergot, 2022).^25^ Although the but-for and the NESS test are rarely discussed in this literature (a notable exception is Baltag et al. (2021)), similar concerns arise in the analysis of joint agency and collective action and, in particular, in questions pertaining to whether individual members are essential (see Belnap and Perloff, 1993; Sergot, 2021).^26^

Let us proceed with briefly reconsidering the example modelled in responsibility game $S_2$ and illustrated in Fig. 2. Notice that, at action profile *a*, the group consisting of Firm 1 and Firm 2 sees to it that the harm obtains. However, neither of these firms individually guarantees that the harm obtains. Hence, each of these firms satisfies the weak NESS condition for $0$ at action profile *a*. Therefore, each firm causally contributes to the realization of $V$.

Notice that we get the deliberative stit by setting ${\mathcal {G}}$ to $\{i\}$ in the definition of a weak NESS condition, and we get the but-for clause by setting ${\mathcal {G}}$ to *N* in that definition. Hence, if an agent deliberatively sees to it that $\varphi$, then she would be a (weak) NESS condition for it; and if an agent is a but-for condition for $\varphi$, then she would also be a (weak) NESS condition for it. It may be helpful to note that this means that the NESS test can be used to argue that criterion (1) of moral responsibility should be *broader* than the criteria characterized by the deliberative stit and the but-for test. After all, Fig. 2 demonstrates that there are cases where an agent is a weak NESS condition for a given state of affairs even though she does not deliberatively guarantee its realization nor is she a but-for condition for its realization.

## Moral Responsibility Under Risk

I follow the standard practice in decision theory and distinguish between three “realms of decision-making under:

1. *Certainty* if each action is known to lead invariably to a specific outcome (the words prospect, stimulus, alternative, etc., are also used).
2. *Risk* if each action leads to one of a set of possible outcomes, each outcome occurring with a known probability.
3. *Uncertainty* if either action or both has as its consequences a set of possible specific outcomes, but where the probabilities of these outcomes are completely unknown or are not even meaningful.” (Luce and Raiffa, 1957, p. 13—emphasis added)^27^

A *responsibility game under risk* extends a responsibility game with probabilities. There are different ways in which one may interpret these probabilities. For the purposes of modelling responsibility, I propose to take these responsibilities to represent *subjective* degrees of beliefs or expectations.^28^ People sometimes form their beliefs in an irrational or epistemically defective way.^29^ Of course, if someone irrationally or unjustifiably falsely believes that her actions will have only good consequences (even though her actions actually bring about harm), then it seems plausible to think that the person is morally responsible. For example, if Firm 1 were to decide to emit toxins themselves because they unjustifiably form the false expectation that the Firm 2 will refrain from emitting toxins, then Firm 1 is still commonly thought to be responsible. Roughly stated, the impression is that Firm 1 should have expected that their emissions would bring about the harm. My discussion of moral responsibility therefore makes most sense if one takes probabilities to represent rational or justified subjective expectations.^30^

To encompass these notions, a responsibility game under risk includes conditional probability distributions. For a given agent *i* and an individual action $a^{}_{i}\in A^{}_{i}$, the conditional probability distribution $P^{}_{i}(-\mid a^{}_{i})$ assigns to each combination of available actions of the others $b^{}_{-i}\in A^{}_{-i}$ its probability $P^{}_{i}(b^{}_{-i}\mid a^{}_{i})\in [0,1]$. These conditional probability distributions allow for the possibility that, for a given agent, the actions of the other agents can probabilistically depend on her own choice of action. These conditional probabilities accommodate evidential decision theory, in general, and causal decision theory in circumstances where the actions of several agents are causally dependent (see (Peterson, 2017, Chapter 9) for an accessible introduction to causal and evidential decision theory).

### **Definition 6**

(Responsibility Game under Risk) A *responsibility game under risk* *R* is a tuple $\langle N, (A^{}_{i}), (P^{}_{i}), V\rangle$, involving a responsibility game $\langle N, (A^{}_{i}), V\rangle$ and for each agent *i* in *N* and each individual action $a^{}_{i}\in A^{}_{i}$ a conditional probability distribution $P^{}_{i}(- \mid a^{}_{i}): A^{}_{-i}\rightarrow [0,1]$.^31^

Given a responsibility game under risk, an action profile, and a state of affairs, it is possible to meticulously represent the fact that an agent (weakly) raises the probability that that state of affairs is realized. To do so in an elegant way, I first present some preliminary definitions. Given a state of affairs $X\subseteq A$ and an action $a^{}_{i}\in A^{}_{i}$, we let $P^{}_{i}(X\mid a^{}_{i}):=\sum \{P^{}_{i}(b^{}_{-i}\mid a^{}_{i})\mid (a^{}_{i}, b^{}_{-i})\in X\}$, which denotes the probability that *X* will be realized if agent *i* performs action $a^{}_{i}$. From a logical perspective, any formula $\varphi$ can be identified with the set of possible worlds in which it is true. That is, given a formula $\varphi$, we can let $\llbracket \varphi \rrbracket _R:=\{a\in A \mid R,a\models \varphi \}$. In particular, notice that $\llbracket 1\rrbracket _R=I$ and $\llbracket 0\rrbracket _R=V$. The above preliminary definition can then be extended to complex formulae: we define $P^{}_{i}(\varphi \mid a^{}_{i}):=P^{}_{i}(\llbracket \varphi \rrbracket _R\mid a^{}_{i})$.

With the help of these auxiliary definitions, I propose that, in a given responsibility game under risk a given action $a^{}_{i}$ *weakly raises the probability* that *X* will be realized if and only if there is no action $b^{}_{i}$ available to her such that the probability that *X* will be realized if agent *i* performs action $b^{}_{i}$ is strictly higher than if she were to perform action $a^{}_{i}$. Or, equivalently, if and only if for every action $b^{}_{i}$ available to her, the probability that *X* will be realized if agent *i* performs action $b^{}_{i}$ is lower or equal to the probability if she were to perform action $a^{}_{i}$.

### **Definition 7**

*(Evaluation Rule: Probability Promoting)* Let *R* be a responsibility game under risk, let $a\in A$ be an action profile, let $i\in N$ be an agent, and let $\varphi$ be a formula. Then, the truth-conditions for $[i\;\textsf{prom}]\varphi$ are given by:

$$\begin{aligned} \begin{array}{lll} R,a\models [i\;\textsf{prom}]\varphi &{} {iff} &{} \text {there is no}\,\, b^{}_{i} \,\,\text {in}\,\, A^{}_{i} \,\,\text {such that}\,\, P^{}_{i}(\varphi \mid b^{}_{i})> P^{}_{i}(\varphi \mid a^{}_{i}).\\ \end{array} \end{aligned}$$

Or, equivalently, $R,a\models [i\;\textsf{prom}]\varphi$ if and only if for all $b^{}_{i}$ it holds that $P^{}_{i}(\varphi \mid b^{}_{i})\le P^{}_{i}(\varphi \mid a^{}_{i})$.^32^

Although objective probabilities have been used in probabilistic models of causation (Vallentyne, 2008), it is surprising that (justified) subjective probability promotion has rarely featured in formal approaches to moral responsibility (exceptions include Broersen, 2011; Brahamand van Hees, 2012; Halpern and Kleiman-Weiner, 2018). Nevertheless, it is natural to model the epistemic state of an agent using subjective probabilities and, in this way, we can express an epistemic version of the agency condition for moral responsibility.

It may be helpful to briefly consider another perspective on this notion of probability promoting. The probabilities of leading to the realization of a given state of affairs can be viewed as inducing an ordering on the available actions of the agents. In particular, we could say that action $a^{}_{i}$ ranks below or equal to action $b^{}_{i}$—in terms of weakly raising the probability of $V$—if and only if $P^{}_{i}(V\mid a^{}_{i})\ge P^{}_{i}(V\mid b^{}_{i})$. Notice that this way of ordering the individual actions yields a linear ordering on $A^{}_{i}$.^33^ From the perspective of this ordering, we can see that a given agent weakly raises the probability that $V$ will be realized if and only if she performs one of the individual actions that is ranked highest in this ordering.

One way to indicate the fruitfulness of the logical approach is by classifying the proposal by Braham and van Hees (2012) in the logical formalism. It is easy to verify that their notion of moral responsibility is characterized as follows. Consider a responsibility game under risk *R*, an action profile *a*, and an agent *i*. Then, Braham and van Hees (2012) propose that agent *i* is morally responsible for $V$ if and only if the conjunction of (1) and (4) obtains. That is, if and only if:

1. $R,a\models [i\;\textsf{caus}]0$: agent *i* causally contributes to the realization of $V$; and
2. $R,a\models \lnot [i\;\textsf{prom}]\lnot [i\;\textsf{caus}]0$: there is an alternative action $b^{}_{i}$ that raises the probability that agent *i* does not causally contribute to the realization of $V$.^34^

### Logical Investigations

I would like to start with examining the logical relations between the idea of promoting the probability that a given state of affairs is realized and the idea of seeing to that it is realized. It is easy to verify that

$$\begin{aligned} \models&\Diamond [i\;\textsf{prom}]\varphi \\ \models&[i\;\textsf{stit}]\varphi \rightarrow [i\;\textsf{prom}]\varphi \\ \not \models&[i\;\textsf{prom}]\varphi \rightarrow [i\;\textsf{stit}]\varphi \end{aligned}$$

The first item establishes that one is always able to promote the probability of a given state of affairs, irrespective of its logical form. The fact that this includes infeasible properties, in particular logical inconsistencies, may seem unsatisfactory. However, for infeasible states of affairs it does not matter what one does, because one’s choice of action does not change the fact that it will not be realized. Indeed, it is impossible to avoid promoting logical inconsistencies. The second and third item indicate that promoting the probability of a given state of affairs is logically weaker than seeing to its realization.^35^

We can derive a couple more intuitive validities once we restrict our analysis to a subclass of the responsibility games under risk. Let us say that a conditional probability distribution $P^{}_{i}$ *has full support* if and only if for every individual action $a^{}_{i}\in A^{}_{i}$ and for every $c^{}_{-i}\in A^{}_{-i}$ it holds that $P^{}_{i}(c^{}_{-i}\mid a^{}_{i})>0$. That is, if an agent’s probability distribution has full support, then, irrespective of her own choice of action, for any possible combination of actions of the others she assigns some non-negative degree of belief in the proposition that they perform that combination. Let *R* be a responsibility game under risk such that agent *i*’s probability distribution has full support. Then, it is easy to verify that:

$$\begin{aligned} R\models&([i\;\textsf{prom}]\varphi \wedge \Diamond \varphi )\rightarrow \langle i\;\textsf{stit}\rangle \varphi \\ R\models&\Diamond [i\;\textsf{stit}]\varphi \rightarrow ([i\;\textsf{prom}]\varphi \leftrightarrow [i\;\textsf{stit}]\varphi ) \end{aligned}$$

The first item establishes that, whenever a given state of affairs is feasible, then promoting the probability of its realization entails that one performs an action that is compatible with its realization. Or, equivalently, under these circumstances, if one performs an action that is incompatible with the realization of that state of affairs, then one is surely not promoting the probability of its realization. The second item shows that in case the agent is able to guarantee the realization of a given state of affairs, then promoting the probability of its realization is logically equivalent to seeing to its realization.

The moral of this brief investigation of the logical relations between stit theory and probability raising is the following. Notice that if an agent deliberatively sees to it that $\varphi$, then she is promoting the probability of its realization. From this perspective, the idea of probability raising can be used to argue that the notion of moral responsibility should be *broader* than the deliberative stit. After all, there are cases where an agent promotes the probability of a given state of affairs even though she does not deliberatively guarantee its realization. Recall that the weak NESS conditions could also be used to argue that the notion of moral responsibility should be *broader* than the deliberative stit. We could thus view the idea of the weak NESS conditions (expressed by the simple criterion (1) $[i\;\textsf{caus}]0$) and the idea of probability raising (expressed by the simple criterion (2) $[i\;\textsf{prom}]0$) as two distinct ways to broaden the notion of moral responsibility.

Let us end with considering some logical relations between the complex criterion for moral responsibility expressed by (5) $[i\;\textsf{prom}][i\;\textsf{caus}]0$ and the criteria expressed in stit theory by the formulas $[i\;\textsf{stit}]0$ and $[i\;\textsf{dstit}]0$. One may ask whether the stit operators entail the agency condition and/or the negative condition associated with the complex criterion expressed by $[i\;\textsf{prom}][i\;\textsf{caus}]0$. It is easy to verify that the following validity and invalidities hold:^36^

$$\begin{aligned} \models&[i\;\textsf{stit}]\varphi \rightarrow [i\;\textsf{prom}][i\;\textsf{caus}]\varphi \\ \not \models&[i\;\textsf{stit}]\varphi \rightarrow \Diamond \lnot [i\;\textsf{prom}][i\;\textsf{caus}]\varphi \\ \not \models&[i\;\textsf{dstit}]\varphi \rightarrow \Diamond \lnot [i\;\textsf{prom}][i\;\textsf{caus}]\varphi \end{aligned}$$

Moreover, once we restrict our analysis to responsibility games under risk where agent *i*’s probability distribution has full support, then the last invalidity becomes valid. Let *R* be a responsibility game under risk where agent *i*’s probability distribution has full support. Then, it is easy to check that:

$$\begin{aligned}R\models [i\;\textsf{dstit}]\varphi \rightarrow \big ([i\;\textsf{prom}][i\;\textsf{caus}]\varphi \wedge \Diamond \lnot [i\;\textsf{prom}][i\;\textsf{caus}]\varphi \big )\end{aligned}$$

Similar validities and invalidities hold between stit theory and the complex criterion for moral responsibility expressed by (7) $[i\;\textsf{prom}]\lnot [i\;\textsf{caus}]\lnot 0$. So, the moral of these last observations is that one can view the complex criteria for moral responsibility expressed by (5) $[i\;\textsf{prom}][i\;\textsf{caus}]0$ and (7) $[i\;\textsf{prom}]\lnot [i\;\textsf{caus}]\lnot 0$ as a third and fourth way to broaden the notion of moral responsibility beyond the deliberative stit.

## The Logical Classification

**Fig. 3:**
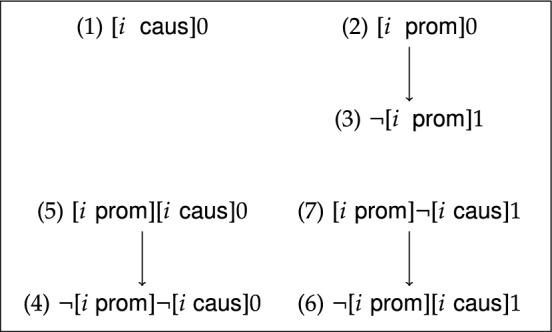
Natural logical relations between the criteria for moral responsibility

Although the seven criteria for moral responsibility (presented in Sect. 2) are *prima facie* distinct, it is an open question whether they truly are *logically* distinct. That is, it is important to investigate the logical relations between the seven criteria for moral responsibility. In this section, I obtain the logical classification for the seven criteria for moral responsibility in scenarios of decision-making under risk. If we let $\varphi$ and $\psi$ depict two agency conditions, then one may ask whether the agency condition expressed by $\varphi$ together with the associated negative condition (formalized by $\Diamond \lnot \varphi$) *logically entail* the agency condition expressed by $\psi$ and its negative condition (formalized by $\Diamond \lnot \psi$). If we represent $\models (\varphi \wedge \Diamond \lnot \varphi )\rightarrow (\psi \wedge \Diamond \lnot \psi )$ by an arrow from $\varphi$ to $\psi$, these logical relations between the seven criteria for moral responsibility can be pictured as in Fig. 3.^37^

### The Three Simple Criteria for Moral Responsibility

**Fig. 4:**
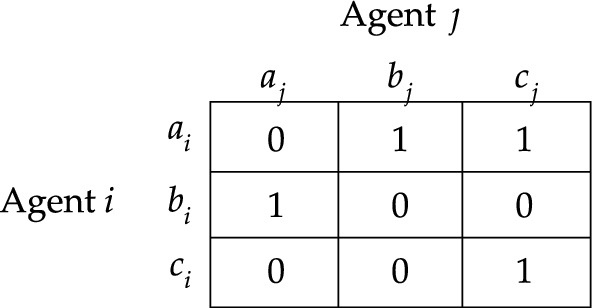
Responsbility game $$S_3$$

Let me start with the more simple criteria of moral responsibility. Given the formal apparatus, it can be proven that there is no logical connection between (1) $[i\;\textsf{caus}]0$, on the one hand, and (2) $[i\;\textsf{prom}]0$ and (3) $\lnot [i\;\textsf{prom}]1$, on the other. To show this, let me present the responsibility game $S_3$ of Fig. 4. It involves two agents ($N=\{i,j\}$) each of whom has three actions available to her (for example, $A^{}_{i}=\{a^{}_{i}, b^{}_{i}, c^{}_{i}\}$), and the state of affairs $V$ is given by the set of action profiles that yield a deontic utility of 0. Consider the responsibility game under risk $R_{3}$ that extends $S_{3}$ with uniform probability distributions.

Notice that agent *i* promotes the probability that $I$ is realized if and only if she performs $a^{}_{i}$. After all, the probability that $I$ is realized is 67% for $a^{}_{i}$ and only 33% for $b^{}_{i}$ and $c^{}_{i}$. In contrast, agent *i* promotes the probability that $V$ is realized if and only if she performs either $b^{}_{i}$ or $c^{}_{i}$. Moreover, notice that, since neither agent *i* nor agent *j* can individually guarantee the realization of $V$, it follows that for any action profile it holds that agent *i* causally contributes to $V$ if and only if that action profile is in $V$ (the same holds for agent *j*). Hence,

$$\begin{aligned} R_{3},(a^{}_{i}, a^{}_{j})&\not \models [i\;\textsf{caus}]0\rightarrow [i\;\textsf{prom}]0\\ R_3,(a^{}_{i}, a^{}_{j})&\not \models [i\;\textsf{caus}]0\rightarrow \lnot [i\;\textsf{prom}]1\\ R_3,(c^{}_{i}, c^{}_{j})&\not \models [i\;\textsf{prom}]0\rightarrow [i\;\textsf{caus}]0\\ R_3,(c^{}_{i}, c^{}_{j})&\not \models \lnot [i\;\textsf{prom}]1\rightarrow [i\;\textsf{caus}]0\\ \end{aligned}$$

Hence, there are no logical connections between (1) and (2) and there are no logical connections between (1) and (3). This is depicted in Fig. 3 by the absence of any implications between, on the one hand, (1) and, on the other hand, (2) and(3).

Let me continue with characterizing the relation between (2) $[i\;\textsf{prom}]0$ and (3) $\lnot [i\;\textsf{prom}]1$. Let us start with investigating whether (3) implies (2). First, notice that the negative condition of (3) is valid (see Sect. 5.1). Second, suppose some morally undesirable state of affairs $V$ results from the interaction of several individuals. Then, we can compare the individual actions available to a given agent in terms of their probability of realizing that morally undesirable state of affairs. Two options immediately come to my mind. First, one may think that an agent can avoid moral responsibility by deciding to not promote the realization of $V$. This is characterized by the negation of (2) $[i\;\textsf{prom}]0$. Second, one may think that an agent can avoid moral responsibility by deciding to promote the probability that $V$ does not obtain. This is characterized by the negation of (3) $\lnot [i\;\textsf{prom}]1$.

**Fig. 5:**
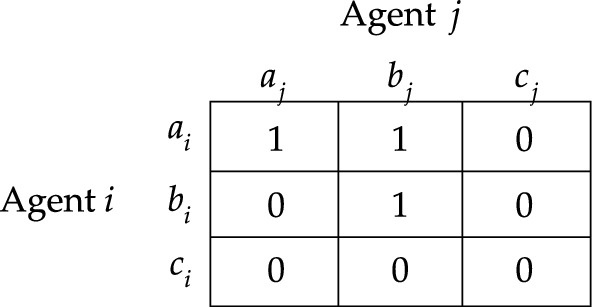
Responsbility game $$S_4$$

Let us indicate the difference in terms of responsibility game $S_4$ presented in Fig. 5. Consider the responsibility game under risk $R_{4}$ that extends $S_{4}$ with uniform probability distributions. Notice that $P^{}_{i}(V\mid a^{}_{i})< P^{}_{i}(V\mid b^{}_{i}) < P^{}_{i}(V\mid c^{}_{i})$ and that $P^{}_{i}(I\mid a^{}_{i})> P^{}_{i}(I\mid b^{}_{i}) > P^{}_{i}(I\mid c^{}_{i})$. According to criterion (2), agent *i* can only be held morally responsible for $V$ if she performs action $c^{}_{i}$. After all, the only way for her to promote the realization of $V$ is by performing $c^{}_{i}$. In contrast, according to criterion (3), agent *i* can be morally criticized if she performs either action $b^{}_{i}$ or action $c^{}_{i}$. That is, she can only avoid moral criticism if she performs action $a^{}_{i}$. As this example illustrates, we have:

$$\begin{aligned}R_4, (b^{}_i,b^{}_j)\not \models \lnot [i\;\textsf{prom}]1\rightarrow [i\;\textsf{prom}]0.\end{aligned}$$

Hence, $R_4$ demonstrates that there is no logical implication from (3) to (2). This is as it should be.

What about the implication from (2) to (3)? The following corollary establishes that the validity of this implication is qualified.^38^

#### **Corollary 1**

*Let* *R* *be a responsibility game under risk, let* *a* *be an action profile, and let* *i* *be an agent. Then, the following are equivalent*

1. $$R,a\models [i\;\textsf{prom}]0\rightarrow \lnot [i\;\textsf{prom}]1$$;
2. *there are actions* $a^{}_{i}$ *and* $b^{}_{i}$ in $A^{}_{i}$ *such that* $P^{}_{i}(V\mid a^{}_{i}) > P^{}_{i}(V\mid b^{}_{i})$;
3. $$R,a\models \Diamond \lnot [i\;\textsf{prom}]0$$.

We, hence, see that the converse implication is valid except when each available action has the same probability of realizing $V$.^39^ Notice that, under these circumstances, each action also has the same probability of realizing $I$. Hence, under these circumstances, the agent promotes the realization of $V$ and promotes the realization of $I$, regardless of her course of action.

From the equivalence of item 1. and item 3. of Corollary 1, it immediately follows that the negative criterion associated with (2) (which is: $\Diamond \lnot [i\;\textsf{prom}]0$) ensures that (2) $[i\;\textsf{prom}]0$ logically entails (3) $\lnot [i\;\textsf{prom}]1$. Hence, Corollary 1 entails that the criterion for moral responsibility expressed by (2) is narrower than the criterion for moral responsibility under (3) (as depicted in Fig. 3).

### The Four Complex Criteria for Moral Responsibility

Are there any other logical relations to be found among the more complex criteria for moral responsibility presented in Sect. 2? Given the formal similarities between the pairs (2)–(3), (5)–(4), and (7)–(6), it should not come as a surprise that a result similar to Corollary 1 holds:^40^

#### **Corollary 2**

*Let* *R* *be a responsibility game under risk, let* *a* *be an action profile, and let* *i* *be an agent. Then, the following are equivalent*

1. $$R,a\models [i\;\textsf{prom}][i\;\textsf{caus}]0\rightarrow \lnot [i\;\textsf{prom}]\lnot [i\;\textsf{caus}]0$$;
2. there are actions $a^{}_{i}$ and $b^{}_{i}$ in $A^{}_{i}$ such that $P^{}_{i}([i\;\textsf{caus}]0\mid a^{}_{i}) > P^{}_{i}([i\;\textsf{caus}]0\mid b^{}_{i})$;
3. $$R,a\models \Diamond \lnot [i\;\textsf{prom}][i\;\textsf{caus}]0$$.

*In addition, the following are equivalent*

1. $$R,a\not \models [i\;\textsf{prom}]\lnot [i\;\textsf{caus}]1\rightarrow \lnot [i\;\textsf{prom}][i\;\textsf{caus}]1$$;
2. *there are actions* $a^{}_{i}$ *and* $b^{}_{i}$ in $A^{}_{i}$ *such that* $P^{}_{i}([i\;\textsf{caus}]1\mid a^{}_{i}) > P^{}_{i}([i\;\textsf{caus}]1\mid b^{}_{i})$;
3. $$R,a\models \Diamond \lnot [i\;\textsf{prom}]\lnot [i\;\textsf{caus}]1$$.

This means that this corollary can be used to characterize the circumstances where (5) $[i\;\textsf{prom}][i\;\textsf{caus}]0$ entails (4) $\lnot [i\;\textsf{prom}]\lnot [i\;\textsf{caus}]0$, and those where (7) $[i\;\textsf{prom}]\lnot [i\;\textsf{caus}]1$ entails (6) $\lnot [i\;\textsf{prom}][i\;\textsf{caus}]1$. In line with the discussion above, it follows that the criterion for moral responsibility expressed by (5) *logically entails* the one expressed by (4), but *not vice versa*. A similar observation applies to (7) and (6).

**Fig. 6:**
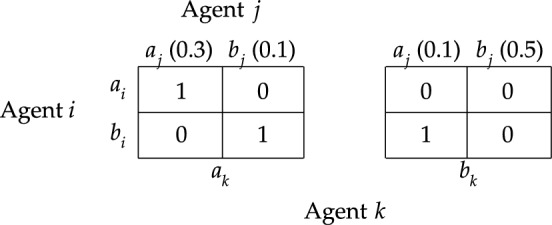
Responsbility game under risk $R_5$, where only the probability distribution of agent *i* is depicted

For reasons of simplicity, I will not dwell on the other logical relations too much here, aside from remarking that there aren’t many logical relations to be found. Let me illustrate this for (2) $[i\;\textsf{prom}]0$ and (5) $[i\;\textsf{prom}][i\;\textsf{caus}]0$ with a formal example. Consider responsibility game under risk $R_{5}$, which is depicted in Fig. 6. Notice that the game involves three agents, namely agent *i*, agent *j*, and agent *k*. Consider agent *i*. It is easy to verify that $[i\;\textsf{caus}]0$ is true at any action profile in $V$ except for $(b^{}_{i},b^{}_{j}, b^{}_{k})$.^41^ Given the degrees of belief of agent *i*, it is easy to verify that $P^{}_{i}(0\mid a^{}_{i}) = 0.7 < 0.8 = P^{}_{i}(0\mid b^{}_{i})$ and $P^{}_{i}([i\;\textsf{caus}]0\mid a^{}_{i}) = 0.7 > 0.3 = P^{}_{i}([i\;\textsf{caus}]0\mid b^{}_{i})$. Hence,

$$\begin{aligned}R_{5},(a^{}_{i},a^{}_{j}, a^{}_{k})\not \models [i\;\textsf{prom}][i\;\textsf{caus}]0\rightarrow [i\;\textsf{prom}]0\end{aligned}$$

and, therefore, $\not \models [i\;\textsf{prom}][i\;\textsf{caus}]0\rightarrow [i\;\textsf{prom}]0$. Moreover,

$$\begin{aligned}R_{5},(b^{}_{i},b^{}_{j},b^{}_{k})\not \models [i\;\textsf{prom}]0\rightarrow [i\;\textsf{prom}][i\;\textsf{caus}]0\end{aligned}$$

and, therefore, $\not \models [i\;\textsf{prom}]0\rightarrow [i\;\textsf{prom}][i\;\textsf{caus}]0$. In other words, the example illustrates that there are no logical relations between proposals (2) and (5).

Finally, it can be shown that there are also no logical relations between any pair of the remaining criteria for moral responsibility. (This follows from Observation 5, which is stated and proved in the appendix.) Hence, we obtain the full classification for the seven criteria for moral responsibility in scenarios of decision-making under risk as depicted in Fig. 3.

## Moral Responsibility Under Uncertainty

Let us proceed with investigating moral responsibility in scenarios of decision-making under uncertainty. If we identify an action with the set of action profiles that are consistent with it, then the ordering of the available actions can be viewed as an ordering over *sets* of action profiles. There are several ways in which we can lift an ordering over action profiles to an ordering over *sets of* action profiles. Here, I will endorse the decision-theoretic notion of admissibility, which combines the sure-thing principle with reasoning by cases (more on this below).

It may help to first discuss a few alternative ways of defining an ordering of the available actions before discussing admissibility. For example, it seems to make sense to focus on either the best or the worst possible outcomes that may result by performing one or the other action. In particular, one could focus on the worst possible outcomes and rank one action above another action if the worst outcome associated with the former is better than the worst outcome associated with the latter. More precisely, if we let $\min (a^{}_{i})$ denote the worst outcome that is compatible with the individual action $a^{}_{i}$, then we could postulate that action $a^{}_{i}$ ranks above action $b^{}_{i}$ if the outcome $\min (a^{}_{i})$ is better than the outcome $\min (b^{}_{i})$. Given that responsibility games include a *binary* deontic utility function, in responsibility games this ordering is equivalent to the following: action $a^{}_{i}$ ranks above action $b^{}_{i}$ if $a^{}_{i}$ guarantees $I$ and $b^{}_{i}$ is compatible with $V$. Alternatively, if one were to focus on the best possible outcomes, then one would get an ordering that amounts to the following in responsibility games: action $a^{}_{i}$ ranks above action $b^{}_{i}$ if $a^{}_{i}$ is compatible with $I$ and $b^{}_{i}$ guarantees $V$.^42^

Alternatively, it is plausible to think that one should base the ordering of the available actions on the ‘average’ outcome associated with them. More precisely, if we define the average of a given action $a^{}_{i}$ by $\text {average}(a^{}_{i}):=\sum \{u(b)\mid b\in A$ and $b^{}_{i}=a^{}_{i}\} / \#\{b\in A\mid b^{}_{i}=a^{}_{i}\}$, then we could postulate that action $a^{}_{i}$ ranks above action $b^{}_{i}$ if $\text {average}(a^{}_{i}) > \text {average}(b^{}_{i})$. It should be noted that this idea can be captured by considering the responsibility game under risk where the probability distributions are given by uniform distributions.

Although the framework can be used to incorporate an alternative ordering, I endorse the decision-theoretic notion of admissibility. I propose that the criterion for moral responsibility characterized by (2) $[i\;\textsf{prom}]0$ can be made precise by requiring the avoidance of inadmissible actions. Admissibility is related to the idea that an agent takes all actions of the other agents into consideration; none is entirely ruled out.

The principle guiding the dominance orderings combines the sure-thing principle and reasoning by cases.^43^ More specifically, an action $a^{}_{i}$ simply dominates $b^{}_{i}$ with respect to the realization of $\varphi$ if and only if $a^{}_{i}$ promotes the realization of $\varphi$ at least as well as $b^{}_{i}$, regardless of what the others do.^44^ More precisely:

### **Definition 8**

*(Dominance)* Let *S* be a responsibility game, let $i\in N$ be an agent, and let $\varphi$ be a formula. Let $a^{}_{i}$ and $b^{}_{i}$ be actions available to agent *i*. Then $a^{}_{i}$ *simply dominates* $b^{}_{i}$ *with respect to* the realization of $\varphi$, notation $a^{}_{i}\succeq _{\varphi }b^{}_{i}$, is defined by:

$$\begin{aligned} \begin{array}{lll} a^{}_i\succeq ^{}_\varphi b^{}_i &{} {iff} &{} \text {for all}\,\, c^{}_{-i}\in A^{}_{-i}\,\, \text {it holds that }\,\, S,(b^{}_{i},c^{}_{-i})\models \varphi \,\,\text {implies }\,\, S,(a^{}_{i},c^{}_{-i})\models \varphi .\\ \end{array} \end{aligned}$$

*Weak dominance* is defined in terms of simple dominance: $a^{}_i\succ ^{}_\varphi b^{}_i$ if and only if $a^{}_i\succeq ^{}_\varphi b^{}_i$ and $b^{}_i\not \succeq ^{}_\varphi a^{}_i$.

Admissibility is defined in terms of dominance. An action $a^{}_i$ in $A^{}_i$ is *admissible* with respect to the realization of $\varphi$ if and only if it is not weakly dominated with respect to the realization of $\varphi$ by any action in $A^{}_i$:

### **Definition 9**

(Admissible Actions) Let *S* be a responsibility game, let $i\in N$ be an agent, and let $\varphi$ be a formula. Then, the set of *i*’s *admissible actions in* *S* *with respect to the realization of* $\varphi$, denoted by $\text {Admissible}_S(i,\varphi )$, is given by

$$\begin{aligned} \begin{array}{lll} \text {Admissible}_S(i,\varphi ) &{} = &{} \{a^{}_i\in A^{}_i\mid \,\, \text {there is no} \,\, b^{}_i\in A^{}_i \,\, \text {such that} \,\, b^{}_i\succ ^{}_\varphi a^{}_i\}.\\ \end{array} \end{aligned}$$

Notice that any formula and any state of affairs can be represented by a binary utility function: given a formula $\varphi$, one could define the associated utility function $u^{}_{\varphi }$ by $u^{}_\varphi (a):=1$ if $S,a\models \varphi$ and 0 otherwise. Then, the definition of simple dominance translates to: $a^{}_{i}\succeq ^{}_\varphi b^{}_{i}$ if and only if for all $c^{}_{-i}\in A^{}_{-i}$ we have $u^{}_\varphi (a^{}_{i},c^{}_{-i})\ge u^{}_\varphi (b^{}_{i},c^{}_{-i})$. This highlights a straightforward connection to the standard weak dominance ordering, which is studied in traditional rational choice theory.^45^

The above considerations can be used to meticulously present the semantics of the promoting-operator in scenarios of decision-making under uncertainty:

### **Definition 10**

*(Evaluation Rule: Possibility Promoting)* Let *S* be a responsibility game, let $a\in A$ be an action profile, let $i\in N$ be an agent, and let $\varphi$ be a formula. Then, the truth-conditions for $[i\;\textsf{prom}]\varphi$ are given by:

$$\begin{aligned} \begin{array}{lll} S,a\models [i\;\textsf{prom}]\varphi &{} {iff} &{} a^{}_{i}\in \text {Admissible}_{S}(i,\varphi ).\\ \end{array} \end{aligned}$$

Or, equivalently, if and only if there is no $b^{}_{i}\in A^{}_{i}$ such that $b^{}_{i}\succ _{\varphi } a^{}_{i}$.

The decision-theoretic concept of admissibility has been used in models of moral responsibility (Tamminga and Duijf, 2017; Duijf and Van De Putte, 2022; Duijf, 2022). These accounts are based on Horty’s (2001) characterization of an agent’s obligations, which relies on this concept of admissibility. Roughly stated, in the context of binary utility functions, a given agent fulfils all of her obligations if and only if she promotes $1$. It is common to assume that moral responsibility requires that some obligation is not fulfilled. Accordingly, these accounts naturally relate to the notion of moral responsibility characterized by (3) $\lnot [i\;\textsf{prom}]1$.

It may be helpful to mention a few logical relations between the deliberative stit modality and the modality for promoting the possibility. It is easy to verify that:

$$\begin{aligned} \models&\Diamond [i\;\textsf{prom}]\varphi \\ \models&[i\;\textsf{stit}]\varphi \rightarrow [i\;\textsf{prom}]\varphi \\ \not \models&[i\;\textsf{prom}]\varphi \rightarrow [i\;\textsf{stit}]\varphi \\ \models&([i\;\textsf{prom}]\varphi \wedge \Diamond \varphi )\rightarrow \langle i\;\textsf{stit}\rangle \varphi \\ \models&\Diamond [i\;\textsf{stit}]\varphi \rightarrow ([i\;\textsf{prom}]\varphi \leftrightarrow [i\;\textsf{stit}]\varphi ) \end{aligned}$$

Recall that these are exactly the same validities as the ones we’ve discussed for the operator for promoting the probability of realizing a given state of affairs. In analogy, one can view these validities as indicating that the promoting operator has a given logical interplay with the stit operator, regardless of whether we’re investigating a scenario of decision-making under risk or under uncertainty.

Let me end this section with some remarks that provide *prima facie* normative justification for admissibility. First, I do believe that if action $a^{}_{i}$ weakly dominates $b^{}_{i}$ with respect to realizing a certain state of affairs $\varphi$, then $a^{}_{i}$ promotes the possibility that $\varphi$ is realized better than $b^{}_{i}$. After all, if a combination of actions of the others leads to the realization of $\varphi$ given $b^{}_{i}$, then $\varphi$ also obtains given $a^{}_{i}$. In particular, when one performs an inadmissible action, then it seems plausible to say that one is not promoting the possibility that $\varphi$ is realized. (Exceptions may arise when certain combinations of actions of the others can be entirely ruled.) Phrased differently, the set of actions by which an agent promotes the possibility that $\varphi$ is realized is a subset of the admissible actions. So, I suggest that the avoidance of inadmissible actions is a good first approximation of promoting the possibility that some state of affairs is realized.

Let me make this intuition more precise. Let us say that a conditional probability distribution $P^{}_{i}$ satisfies *probabilistic independence* if for all individual actions $a^{}_{i}$ and $b^{}_{i}$ in $A^{}_{i}$ and for every $c^{}_{-i}$ in $A^{}_{-i}$ it holds that $P^{}_{i}(c^{}_{-i}\mid a^{}_{i})= P^{}_{i}(c^{}_{-i}\mid b^{}_{i})$. That is, the conditional probability that the other agents perform $c^{}_{-i}$ does not depend on agent *i*’s choice of action. (All the examples discussed in this paper satisfy this criterion.)

Consider the assumptions that the probability distributions satisfy probabilistic independence and that they have full support. Then, any action that (weakly) raises the probability that a given state of affairs is realized is also admissible with respect to that state of affairs.^46^ More precisely:

### **Observation 1**

*Let* *R* *be a responsibility game under risk, let* $i\in N$ *be an agent, and let* $\varphi$ *be a formula. Let* $a^{}_i$ *and* $b^{}_i$ *be individual actions available to agent* *i*. *Assume that the probability distribution* $P^{}_{i}$ *satisfies probabilistic independence and has full support. Then*:

- *if there is no* $b^{}_{i}$ *in* $A^{}_{i}$ *such that* $P^{}_i(\varphi \mid b^{}_i) > P^{}_i(\varphi \mid a^{}_i)$, *then* $a^{}_{i}$ *is admissible with respect to* $\varphi$.

Notice that this observation covers the idea that one should base the ordering of the available actions on the average outcome associated with those actions. Therefore, if an action $a^{}_{i}$ is such that there is no action $b^{}_{i}$ such that $\text {average}(b^{}_{i})> \text {average}(a^{}_{i})$, then that action is admissible with respect to $1$.

In relation to the assumption of probabilistic independence, it should be noted that the admissibility theory makes most sense in cases where the agents’ actions are causally independent (see the illuminating discussion by Horty (2001, Sects. 4.1 and 4.4).^47^ After all, the legitimacy of weak dominance hinges on the fact that it makes sense to reason by cases, where these cases are identified with the available combinations of actions of the others. Therefore, in scenarios of decision-making under uncertainty that violate causal independence, the normative appeal of dominance theory is weakened.

One of the biggest drawbacks of admissibility is the incompleteness of weak dominance. Some even argue that weak dominance virtually never obtains in real-world scenarios. This incompleteness would trump the idea that admissibility is a sufficient condition for avoiding moral responsibility. Refining the notion of admissibility to deal with these challenges in cases of decision-making under uncertainty is a formidable research task that cannot be pursued here.

## Local Reductions

I propose to investigate the conditions under which the complex criteria for moral responsibility collapse to one of the simpler criteria for moral responsibility. In the following, I postulate three natural subclasses of cases and demonstrate that the four complex criteria for moral responsibility can be logically reduced to the three simple criteria when our attention is restricted to any of these subclasses.

To specify these natural subclasses, we need to introduce two additional concepts. Let us say that in a responsibility game under risk *R* a given agent *i* satisfies *simple causation* for a given state of affairs expressed by $\varphi$ if agent *i* makes a causal contribution to the realization of $\varphi$ if and only if she deliberatively sees to it that $\varphi$ or her action is a but-for condition for $\varphi$. In other words, agent *i* satisfies simple causation for $\varphi$ if $R\models [i\;\textsf{caus}]\varphi \leftrightarrow ([i\;\textsf{dstit}]\varphi \vee [i\;\textsf{butfor}]\varphi )$.

Lastly, let us say that in a responsibility game under risk *R* a given agent *i*’s actions are *totally ordered* for $\varphi$ if for any individual actions $a^{}_{i}$ and $b^{}_{i}$ we have $a^{}_{i}\succeq _{\varphi }b^{}_{i}$ or $b^{}_{i}\succeq _{\varphi }a^{}_{i}$. In particular, if an agent’s actions are totally ordered for $\varphi$, then any action that is admissible with respect to the realization of $\varphi$ simply dominates every other available action. Since incompleteness is one of the most crucial problem with the dominance theory, it is important to remark that the dominance ordering is complete if the available actions are totally ordered.

**Fig. 7:**
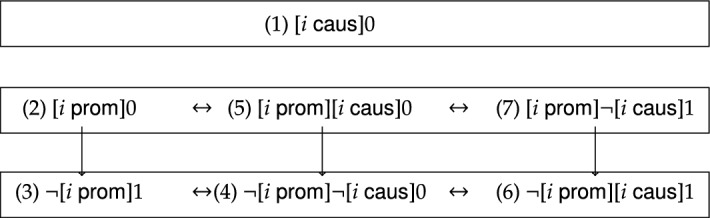
Local reductions of the criteria for moral responsibility

The specific subclasses that I investigate are the following: Cases of decision-making under uncertainty (Sect. 8.1); cases of decision-making under risk where the probability distributions satisfy probabilistic independence and have full support, and in addition either the agent’s actions are totally ordered for $0$ (Sect. 8.2) or the agent satisfies simple causation for $0$ and $1$ (Sect. 8.3). The central results can be illustrated in a diagram. Let ${\mathcal {C}}$ denote any of the above subclasses. Then, if we let an arrow from the agency condition expressed by $\varphi$ to the one expressed by $\psi$ represent the fact that ${\mathcal {C}}\models (\varphi \wedge \Diamond \lnot \varphi ) \rightarrow (\psi \wedge \Diamond \lnot \psi )$, the central results can be pictured as in Fig. 7.^48^

Let me mention some general implications of these results (more specific philosophical reflections are relegated to the subsections below). These results shed some doubt on the justification for considering any of the complex criteria for moral responsibility. After all, if one wishes to defend a particular complex criteria for moral responsibility, then this requires the consideration of complicated cases outside the three natural subclasses of cases postulated above. In the subsections below, I reflect on whether it is reasonable to do so for each of the particular subclasses.

In particular, these results demonstrate that the theory of moral responsibility by Braham and van Hees (2012) is actually very close to the theory characterized by the conjunction of (1) $[i\;\textsf{caus}]0$ and (3) $\lnot [i\;\textsf{prom}]1$. That is, in a considerable set of circumstances, their theory is logically equivalent to the latter conjunction. Moreover, recall that the theories of moral responsibility in (Tamminga and Duijf, 2017; Duijf and Van De Putte, 2022; Duijf, 2022) (which are based on Horty’s (2001) account of obligations) include the criterion characterized by (3) $\lnot [i\;\textsf{prom}]1$. Hence, although the latter theories omit criteria (1), this demonstrates that they are close relatives of the theory of moral responsibility proposed by Braham and van Hees.

Second, on a more positive reading, these results indicate that some criteria for moral responsibility are logically equivalent in a wide class of cases. In other words, under certain specific circumstances, it is logically impossible to distinguish between some of the criteria for moral responsibility. Even though the reasons for holding someone responsible may vary for these criteria for moral responsibility, they do not differ in their judgement of whether someone is morally responsible in these natural subclasses of cases. These results hence indicate a considerable scope of agreement.

Third, we may wonder about the practical significance of adopting one or the other criterion for moral responsibility. If it turns out that reality predominantly involves cases that fall within these three subclasses, then one could conclude that nothing much hinges on how moral responsibility is characterized. In other words, if the cases outside these three subclasses are exotic and sparse, then we need not bother too much about the details of the characterization of moral responsibility.

The flip-side of the above remarks is that these results provide guidance to a proponent of one of the complex criteria for moral responsibility. A convincing argument for adopting one of the complex criteria for moral responsibility must highlight the prevalence and importance of cases outside these three subclasses.

Finally, I propose that these results support a kind of *robustness analysis*. We can think of the criteria for moral responsibility as underlying different theories of responsibility that tell us who is responsible for what. Suppose one is investigating a particular case study and wants to show that a particular individual cannot be held responsible. How does one demonstrate that a particular individual cannot be held responsible? One possibility is to endorse one or several of these criteria for moral responsibility and then show that the particular individual does not meet those criteria. But, how can one show that these judgements of moral responsibility do not depend on disputable details of the endorsed theory of responsibility? My results demonstrate that if the case study falls within one of the three postulated subclasses, then one can prove that a particular individual cannot be held morally responsible by showing that that individual is not morally responsible according to the broadest criteria for moral responsibility. After all, if the individual is not morally responsible according to the broadest criteria for moral responsibility, then she will not be morally responsible according to *any* of these criteria for moral responsibility. The statement that the individual is not morally responsible would then be robustly true.

### Decision-Making Under Uncertainty

I prove that for decision-making under uncertainty, the four complex criteria for moral responsibility can be reduced to the three simple criteria (Corollary 3). In contrast to the scenarios of decision-making under risk, this fact holds without qualification; it is entailed by the following, more general, theorem and observation:

#### **Theorem 1**

*Let* *S* *be a responsibility game, let* $i\in N$ *be an agent, and let* $\varphi$ *be a formula. Let* $a^{}_i$ *and* $b^{}_i$ *be individual actions available to agent* *i*. *Then*:

$$\begin{aligned} \begin{array}{lll} a^{}_i \succeq _{\varphi } b^{}_i &{} iff &{} a^{}_i \succeq _{[i\;\textsf{caus}]\varphi } b^{}_i.\\ \end{array} \end{aligned}$$

*As a consequence,* $a^{}_{i}$ *is admissible with respect to* $\varphi$ *if and only if* $a^{}_{i}$ *is admissible with respect to* $[i\;\textsf{caus}]\varphi$. *That is*:

$$\begin{aligned} \begin{array}{lll} \text {Admissible}_{S}(i,\varphi ) &{} = &{} \text {Admissible}_{S}(i,[i\;\textsf{caus}]\varphi ).\\ \end{array} \end{aligned}$$

#### **Observation 2**

*Let* *S* *be a responsibility game, let* $i\in N$ *be an agent, and let* $\varphi$ *be a formula*. *Let* $a^{}_i$ *and* $b^{}_i$ *be individual actions available to agent* *i*. *Then*:

$$\begin{aligned} \begin{array}{lll} a^{}_i \succeq _{\varphi } b^{}_i &{} iff &{} b^{}_i \succeq _{\lnot \varphi } a^{}_i.\\ \end{array} \end{aligned}$$

#### **Corollary 3**

*Let* *S* *be a responsibility game and let* $i\in N$ *be an agent. Then,*

$$\begin{aligned} \begin{array}{lll} S\models [i\;\textsf{prom}]0\leftrightarrow [i\;\textsf{prom}][i\;\textsf{caus}]0\\ S\models \lnot [i\;\textsf{prom}]1\leftrightarrow \lnot [i\;\textsf{prom}][i\;\textsf{caus}]1\\ S\models [i\;\textsf{prom}]0\leftrightarrow [i\;\textsf{prom}]\lnot [i\;\textsf{caus}]1\\ S\models \lnot [i\;\textsf{prom}]1\leftrightarrow \lnot [i\;\textsf{prom}]\lnot [i\;\textsf{caus}]0.\\ \end{array} \end{aligned}$$

The fact that these equivalences hold without qualification sheds some doubt on the motivations for considering any of the four more complex criteria for moral responsibility. After all, the result indicates that we can make do with the three simpler criteria for moral responsibility whenever we’re considering a scenario of decision-making under uncertainty. From this perspective, if one would like to argue that the simpler criteria do not suffice and that one of the more complex criteria needs to be endorsed instead, then that argument is likely to rely on sophisticated scenarios that include probability assessments.

### Total Ordering

One may wonder whether there are any logical relations between scenarios of decision-making under risk and scenarios of decision-making under uncertainty (in addition to Observation 1). In the following, I prove that, under certain circumstances, a given action is admissible with respect to realizing a given state of affairs if and only if that action promotes the probability of its realization. One of the crucial circumstances are those where the agent’s actions are totally ordered for that state of affairs. It follows from Corollary 3 that, under these circumstances, the four complex criteria for moral responsibility can be reduced to the simple criteria for moral responsibility (see Corollary 4).

Consider the assumptions that the probability distributions satisfy probabilistic independence and have full support, and that the agent’s actions are totally ordered with respect to the realization of a given state of affairs. Then, that agent performs an action that is admissible with regard to the realization of that state of affairs *if and only if* the agent promotes the probability of its realization. More formally:

#### **Observation 3**

*Let R be a responsibility game under risk, let* $i\in N$ *be an agent, and let* $\varphi$ *be a formula*. *Suppose that agent* *i*’*s probability distribution has full support and satisfies probabilistic independence. Assume that agent i*’*s actions are totally ordered for* $\varphi$. *Let* $a^{}_{i}$ and $b^{}_{i}$ *be individual actions available to agent* *i*. *Then*:

$$\begin{aligned} \begin{array}{lll} a^{}_i \succeq _{\varphi } b^{}_i &{} iff &{} P^{}_{i}(\varphi \mid a^{}_i) \ge P^{}_{i}(\varphi \mid b^{}_i).\\ \end{array} \end{aligned}$$

*Consequently, any action is admissible in the responsibility game S if and only if that action promotes the probability in the responsibility game under risk* *R*.

In light of Theorem 1, it follows that, under these circumstances, the four complex criteria for moral responsibility can be reduced to the three simple criteria for moral responsibility:

#### **Corollary 4**

*Let R be a responsibility game under risk, let* $i\in N$ *be an agent, and let* $\varphi$ *be a formula. Suppose that agent* *i*’*s probability distribution has full support and satisfies probabilistic independence. Assume that agent* *i*’*s actions are totally ordered for* $1$. *Then*:

$$\begin{aligned} \begin{array}{lll} R\models \lnot [i\;\textsf{prom}]1\leftrightarrow \lnot [i\;\textsf{prom}]\lnot [i\;\textsf{caus}]0.\\ R\models [i\;\textsf{prom}]0\leftrightarrow [i\;\textsf{prom}][i\;\textsf{caus}]0.\\ R\models \lnot [i\;\textsf{prom}]1\leftrightarrow \lnot [i\;\textsf{prom}][i\;\textsf{caus}]1.\\ R\models [i\;\textsf{prom}]0\leftrightarrow [i\;\textsf{prom}]\lnot [i\;\textsf{caus}]1.\\ \end{array} \end{aligned}$$

Let me emphasize some repercussions of this result. First, it should be noted that an agent’s actions are often totally ordered. For example, one could study whether a given individual is morally (co-)responsible for climate change. To do so, one could consider various polluting activities in virtue of their contribution to global warming. Under the assumption that the consequences of global warming will be irreversible if pollution exceeds some threshold, the agent’s actions are totally ordered with regard to global warming. After all, it seems plausible that any two activities can be compared with respect to how much pollution they produce and, therefore, the activity that produces less pollution will simply dominate the other activity with regard to avoiding global warming.

**Fig. 8:**
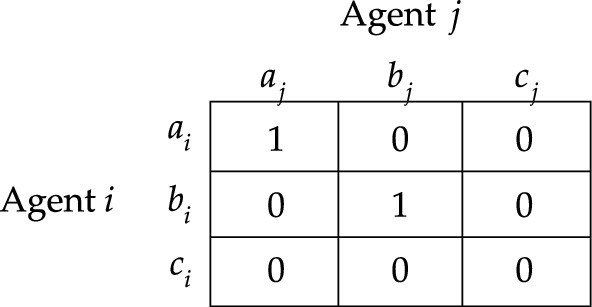
Responsbility game $$S_6$$

What would a responsibility game under risk that violates the total ordering condition look like? Let us briefly reflect on the structure of decision problems where $\succeq _{1}$ fails to be total ordering. In such a case, there exist two incomparable actions, say $a^{}_{i}$ and $b^{}_{i}$ in $A^{}_{i}$. In particular, whether $a^{}_{i}$ or $b^{}_{i}$ produces the best outcome depends on the actions of the others. For example, this includes scenarios where there are two incomparable *admissible* actions. To illustrate the logical possibility of two incomparable admissible actions, consider responsibility game $S_6$ presented in Fig. 8. Notice that $\text {Admissible}(i,1)=\{a^{}_{i}, b^{}_{i}\}$ and $\text {Admissible}(j,1)=\{a^{}_{j}, b^{}_{j}\}$. Informally, one could say that whether agent *i* should choose $a^{}_{i}$ or $b^{}_{i}$ depends on whether agent *j* chooses $a^{}_{j}$ or $b^{}_{j}$, respectively.

These cases are typically referred to as *coordination problems*. Notice that there are coordination problems where the four complex criteria for moral responsibility still reduce to the simple criteria for moral responsibility. For instance, it is easy to verify that this is the case for any responsibility game under risk based on responsibility game $S_{6}$ (see Fig. 8) where the probability distributions satisfy probabilistic independence and have full support. In future work, it would be interesting to characterize the coordination problems where these reductions are impossible.

### Simple Causation

In the following, I show that, under certain circumstances, the four complex criteria for moral responsibility can be reduced to the simple criteria for moral responsibility (see Corollary 5). One of the conditions is that the agent satisfies simple causation for $\varphi$. That is, that the situation is such that the agent causally contributes to the realization of $\varphi$ if and only if she deliberatively sees to it that $\varphi$ or her action is a but-for condition for $\varphi$.

This result follows from a more general theorem. Consider the assumptions that the probability distributions satisfy probabilistic independence and have full support, and that the agent satisfies simple causation for a given state of affairs $\varphi$. Then, that agent performs an action that (weakly) raises the probability of $\varphi$ *if and only if* the agent performs an action that (weakly) raises the probability that she causally contributes to $\varphi$. More formally:

#### *Theorem 2*

*Let* *R* *be a responsibility game under risk, let* *i* *in* *N* *be an agent, and let* $\varphi$ *be a formula. Let* $a^{}_{i}$ *and* $b^{}_{i}$ *in* $A^{}_{i}$ *be actions available to agent* *i*. *Suppose that agent* *i*’*s* *probability distribution has full support and satisfies probabilistic independence. Assume that agent* *i* *satisfies simple causation for* $\varphi$. *Then*:

$$\begin{aligned} \begin{array}{lll} P^{}_{i}(\varphi \mid a^{}_{i}) \ge P^{}_{i} (\varphi \mid b^{}_{i}) &{} iff &{} P^{}_{i}([i\;\textsf{caus}]\varphi \mid a^{}_{i}) \ge P^{}_{i} ([i\;\textsf{caus}]\varphi \mid b^{}_{i}).\\ \end{array} \end{aligned}$$

#### **Corollary 5**

*Let* *R* *be a responsibility game under risk and let* *i* *in* *N* *be an agent. Suppose that agent* *i*’*s* *probability distribution has full support and satisfies probabilistic independence. Assume that agent* *i* *satisfies simple causation for* $0$. *Then*:

$$\begin{aligned} \begin{array}{lll} R\models \lnot [i\;\textsf{prom}]1\leftrightarrow \lnot [i\;\textsf{prom}]\lnot [i\;\textsf{caus}]0\\ R \models [i\;\textsf{prom}]0\leftrightarrow [i\;\textsf{prom}][i\;\textsf{caus}]0. \\ \end{array} \end{aligned}$$

*Assume that agent* *i* *satisfies simple causation for* $1$. *Then*:

$$\begin{aligned} \begin{array}{lll} R \models \lnot [i\;\textsf{prom}]1\leftrightarrow \lnot [i\;\textsf{prom}][i\;\textsf{caus}]1\\ R \models [i\;\textsf{prom}]0\leftrightarrow [i\;\textsf{prom}]\lnot [i\;\textsf{caus}]1. \\ \end{array} \end{aligned}$$

Let me explore some ramifications of this result. First, although the assumption that the agent satisfies simple causation may seem strong, it is important to recognize that *every two-player game* satisfies this assumption. Hence, in any two-player responsibility game under risk, where the probability distrubution satisfies the stated conditions, it holds that the four complex criteria for moral responsibility are equivalent to one of the simpler criteria for moral responsibility.

Second, one might be interested in finding the one true theory of moral responsibility—or, at least, the appropriate theory of moral responsibility for a given scenario. From this perspective, these results indicate that for a natural class of cases we can make do with the three simpler criteria for moral responsibility. Or, equivalently, if one wants to argue that the simpler criteria do not suffice and that one of the more complex criteria needs to be endorsed instead, then that argument is likely to rely on complex scenarios involving more than two individuals.

From this perspective, let us briefly reflect on cases where the probability distributions lack full support. It may be helpful to add that in two-player scenarios the three simpler criteria may, then, not suffice in these cases. Or, equivalently, if a player can entirely rule out particular actions of the other, then it may be that one of the more complex criteria for moral responsibility is not equivalent to any of the simple criteria for moral responsibility.^49^ However, probability-zero events are highly controversial, especially when these events concern the actions of others. After all, how could one completely exclude the possibility that another agent performs a given action *while* assuming that that action is available to her?

What about cases where the probability distributions violate probabilistic dependence? The violation of probabilistic dependence makes most sense in cases of sequential decision-making. These are cases in which the first agent’s action may causally influence the second agent’s decision. Arguably, the weak NESS conditions fail to capture causality in these circumstances. In the last decades, structural-equations models seem to have become the dominant paradigm for dealing with cases of causal dependence (see, among others, Chockler and Halpern, 2004; Halpern and Pearl, 2005; Woodward and Hitchcock, 2003). So, in any case, it seems that the violation of probabilistic independence requires more complex models of causation.

Finally, Theorem 2 can be taken to show that whenever causal contributions are modelled by the disjunction of deliberative stit and the but-for conditions, then the four more complex criteria for moral responsibility can be reduced to the three simpler criteria. This indicates that whether these more complex criteria for moral responsibility are logically distinguishable from the more simple criteria *depends on* how one models causal contributions. This should not come as a surprise, but the result neatly demonstrates this logical dependency.

## Discussion

I have presented a logical study of criteria for moral responsibility for outcomes. The main contributions of this paper are threefold. In the first part, I have introduced a simple multi-modal logical language and demonstrated that it can express various alternative criteria for moral responsibility. For simplicity’s sake, in this paper, I have focused on the seven most simple criteria for moral responsibility:

1. $$[i\;\textsf{caus}]0$$;
  An agent can only be held morally responsible for a given state of affairs if she causally contributed to its realization.
2. $$[i\;\textsf{prom}]0$$
  An agent can only be held morally responsible for a given state of affairs if she promoted its realization.
3. $$\lnot [i\;\textsf{prom}]1$$;
  An agent can only be held morally responsible for a given state of affairs if it is not the case that she promoted that it would not obtain.
4. $$\lnot [i\;\textsf{prom}]\lnot [i\;\textsf{caus}]0$$;
  An agent can only be held morally responsible for a given state of affairs if it is not the case that she promotes that she does not causally contribute to its realization.
5. $$[i\;\textsf{prom}][i\;\textsf{caus}]0$$;
  An agent can only be held morally responsible for a given state of affairs if she promotes that she herself causally contributes to its realization.
6. $$\lnot [i\;\textsf{prom}][i\;\textsf{caus}]1$$;
  An agent can only be held morally responsible for a given state of affairs if it is not the case that she promotes that she causally contributes to avoiding its realization.
7. $$[i\;\textsf{prom}]\lnot [i\;\textsf{caus}]1$$;
  An agent can only be held morally responsible for a given state of affairs if she promotes it is not the case that she causally contributes to avoiding its realization.

One of the central benefits of using this logical language, as opposed to using natural language, is that it helps us avoid ambiguity and it enables us to meticulously characterize these different criteria for moral responsibility.

In the second part, I have illustrated how we can provide truth-conditions for these criteria for moral responsibility using responsibility games. The truth-conditions for the $[i\;\textsf{prom}]$ operator depend on whether it is interpreted in a scenario of decision-making under risk or under uncertainty. These truth-conditions enable us to study moral responsibility more rigorously and more generally.

In the third part, the logical apparatus facilitated the investigation of the logical relations between these various criteria for moral responsibility (summarized in Fig. 3). In particular, my central results demonstrate that, under some circumstances, the four complex criteria for moral responsibility can be reduced to the three simple criteria for moral responsibility (summarized in Fig. 7).

I would like to finish with highlighting one interesting extension of this logical framework. So far, I have only considered cases where there is a single morally relevant state of affairs. However, it is of vital interest to expand to cases where several states of affairs are morally relevant. For example, consider the case of global warming. Following up on the discussion in Sect. 8.2, it is plausible to assume that any activity that produces some pollution does not promote that global warming is avoided. Hence, at least under some criteria for moral responsibility, any agent that produces some pollution is morally responsible for global warming. This may be too simplistic. For example, it may be that some parents can only support their family by driving to their work. It seems plausible that in these scenarios there are two competing morally relevant states of affairs that require consideration. The first involves global warming and the second involves supporting one’s family. My framework naturally allows for an extension to cases involving multiple morally relevant states of affairs. The study of these more complex cases is left to future work.

## Appendix: Omitted Proofs

### Responsibility Games Under Risk

#### Observation 4

*Let* *R* *be a responsibility game under risk, let* *i* *be an agent, and let* $\varphi$ *be a formula. Then, the following are equivalent*

1. for every *a* in *A* it holds that $R,a\models [i\;\textsf{prom}]\lnot \varphi \rightarrow \lnot [i\;\textsf{prom}]\varphi$;
2. there are actions $a^{}_{i}$ and $b^{}_{i}$ in $A^{}_{i}$ such that $P^{}_{i}(\varphi \mid a^{}_{i}) > P^{}_{i}(\varphi \mid b^{}_{i})$;
3. $$R,a\models \Diamond \lnot [i\;\textsf{prom}]\lnot \varphi$$.

#### Proof

(1. $\Rightarrow$ 2.) Note that $\models \Diamond [i\;\textsf{prom}]\lnot \varphi$ (see the first validity in Sect. 5.1). Hence, there is an action profile *b* such that $R,b\models [i\;\textsf{prom}]\lnot \varphi$. By assumption, we get $S,b\models \lnot [i\;\textsf{prom}]\varphi$. Therefore, there is a $a^{}_{i}$ in $A^{}_{i}$ such that $P^{}_{i}(\varphi \mid a^{}_{i}) > P^{}_{i}(\varphi \mid b^{}_{i})$, as desired.

(2. $\Rightarrow$ 1.) Let action profiles *a* and *b* be such that $a^{}_{i}$ and $b^{}_{i}$ together satisfy the condition stated in 2. Take any action profile *c*. Suppose $S,c\models [i\;\textsf{prom}]\lnot \varphi$. Then, $P^{}_{i}(\lnot \varphi \mid c^{}_{i}) \ge P^{}_{i}(\lnot \varphi \mid b^{}_{i})$. Hence, $P^{}_{i}(\varphi \mid c^{}_{i}) \le P^{}_{i}(\varphi \mid b^{}_{i}) < P^{}_{i}(\varphi \mid a^{}_{i})$. Therefore, $S,c\models \lnot [i\;\textsf{prom}]\varphi$, as desired.

(2. $\Rightarrow$ 3.) Let action profiles *a* and *b* be such that $a^{}_{i}$ and $b^{}_{i}$ together satisfy the condition stated in 2. Then, $P^{}_{i}(\lnot \varphi \mid a^{}_{i})< P^{}_{i}(\lnot \varphi \mid b^{}_{i})$. Hence, $R,a\models \lnot [i\;\textsf{prom}]\lnot \varphi$, as desired.

(3. $\Rightarrow$ 2.) Let action profile *a* be such that $R,a\models \lnot [i\;\textsf{prom}]\lnot \varphi$. Then, there is an action profile *b* such that $P^{}_{i}(\lnot \varphi \mid a^{}_{i}) < P^{}_{i}(\lnot \varphi \mid b^{}_{i})$. Hence, $P^{}_{i}(\varphi \mid a^{}_{i}) > P^{}_{i}(\varphi \mid b^{}_{i})$, as desired. $\square$

#### Observation 5

*There are no logical relations between the seven notions of moral responsibility except for the ones mentioned in Corollaries* 1*and* 2.

#### Proof

Consider responsibility game under risk $R_5$, which is depicted in Fig. 6. Recall that $[i\;\textsf{caus}]0$ holds at any action profile in $V$ except for $(b^{}_{i}, b^{}_{j}, b^{}_{k})$. Given the degrees of belief of agent *i*, we can easily calculate that $P^{}_{i}(0\mid a^{}_{i}) = 0.7 < 0.8 = P^{}_{i}(0\mid b^{}_{i})$, $P^{}_{i}(1\mid a^{}_{i}) = 0.3 > 0.2 = P^{}_{i}(1\mid b^{}_{i})$, $P^{}_{i}([i\;\textsf{caus}]0\mid a^{}_{i}) = 0.7 > 0.3 = P^{}_{i}(\lnot [i\;\textsf{caus}]0\mid b^{}_{i})$, and $P^{}_{i}(\lnot [i\;\textsf{caus}]0\mid a^{}_{i}) = 0.3 < 0.7 = P^{}_{i}(\lnot [i\;\textsf{caus}]0\mid b^{}_{i})$ Hence,

$$\begin{aligned} R_{5}\models [i\;\textsf{prom}]0\leftrightarrow \lnot [i\;\textsf{prom}]1, \end{aligned}$$

and

$$\begin{aligned} R_{5}\models [i\;\textsf{prom}][i\;\textsf{caus}]0\leftrightarrow \lnot [i\;\textsf{prom}]\lnot [i\;\textsf{caus}]0. \end{aligned}$$

Therefore, the example illustrates that there are no logical relations between the pairs (2) and (4); (3) and (5); and (3) and (4).

Moreover, notice that $[i\;\textsf{caus}]1$ holds at every action profile in $I$. Therefore $R_{5}\models 1\leftrightarrow [i\;\textsf{caus}]1$. Hence,

$$\begin{aligned} R_{5}\models [i\;\textsf{prom}]0\leftrightarrow [i\;\textsf{prom}]\lnot [i\;\textsf{caus}]1, \end{aligned}$$

and

$$\begin{aligned} R_{5}\models \lnot [i\;\textsf{prom}]1\leftrightarrow \lnot [i\;\textsf{prom}][i\;\textsf{caus}]1. \end{aligned}$$

Therefore, the example illustrates that there are no logical relations between the pairs where the first component is (4) or (5) and the second component is (6) or (7).

An example similar to $R_{5}$ can be used to indicate that the other logical relations are absent. Consider the responsibility game under risk $S^{*}_{5}$ that results from $R_{5}$ by only amending the set $I$ to $I^{*}:=A\setminus I$. It is easy to verify that $S^{*}_{5}$ illustrates that there are no logical relations between the pairs where the first component is (2) or (3) and the second component is (6) or (7). $\square$

#### Theorem 2

*Let* *R* *be a responsibility game under risk, let* *i* *in* *N* *be an agent, and let* $\varphi$ *be a formula. Let* $a^{}_{i}$ *and* $b^{}_{i}$ *in* $A^{}_{i}$ *be actions available to agent* *i*. *Suppose that agent* *i*’*s probability distribution has full support and satisfies probabilistic independence. Assume that agent* *i* *satisfies simple causation for* $\varphi$. *Then*:

$$\begin{aligned} \begin{array}{lll} P^{}_{i}(\varphi \mid a^{}_{i}) \ge P^{}_{i} (\varphi \mid b^{}_{i}) &{} \textit{iff} &{} P^{}_{i}([i\;\textsf{caus}]\varphi \mid a^{}_{i}) \ge P^{}_{i} ([i\;\textsf{caus}]\varphi \mid b^{}_{i}). \\ \end{array} \end{aligned}$$

#### Proof

Recall that $$\models \varphi \rightarrow \lnot [i\;\textsf{caus}]\lnot \varphi$$.

($\Rightarrow$) Consider any action $a^{}_{-i}\in A^{}_{-i}$. We first consider two basic cases, before dealing with the most complex case. First, assume $S,(a^{}_{i},a^{}_{-i})\models \Box \varphi$. Then, $S,(a^{}_{i},a^{}_{-i})\models \Box \lnot [i\;\textsf{caus}]\varphi$. Hence $P^{}_{i}([i\;\textsf{caus}]\varphi \mid a^{}_{i}) = 0 = P^{}_{i} ([i\;\textsf{caus}]\varphi \mid b^{}_{i})$. Second, assume $S,(a^{}_{i},a^{}_{-i})\models [i\;\textsf{stit}]\varphi \wedge \Diamond \lnot \varphi$. Then, $S,(a^{}_{i},a^{}_{-i})\models [i\;\textsf{stit}][i\;\textsf{caus}]\varphi$. Hence, $P^{}_{i}([i\;\textsf{caus}]\varphi \mid a^{}_{i}) = 1 \ge P^{}_{i} ([i\;\textsf{caus}]\varphi \mid b^{}_{i})$.

In the remainder of the proof we assume that $S,(a^{}_{i},a^{}_{-i})\models \lnot [i\;\textsf{stit}]\varphi \wedge \Diamond \lnot \varphi$. Notice that this entails that for every $c^{}_{-i}\in A^{}_{-i}$ it holds that $S,(a^{}_{i},c^{}_{-i})\models \lnot [i\;\textsf{stit}]\varphi \wedge \Diamond \lnot \varphi$. Given our assumption on *S*, this entails that for any $c^{}_{-i}\in A^{}_{-i}$ we have $S,(a^{}_{i},c^{}_{-i})\models [i\;\textsf{caus}]\varphi \leftrightarrow [i\;\textsf{butfor}]\varphi$. Hence,

$$\begin{aligned} P^{}_{i}([i\;\textsf{caus}]\varphi \mid a^{}_{i})&= P^{}_{i}(\varphi \wedge \lnot [N-i\;\textsf{stit}]\varphi \mid a^{}_{i}). \end{aligned}$$

Moreover, since for any $c^{}_{-i}\in A^{}_{-i}$ it holds that $S,(a^{}_{i},c^{}_{-i})\models [N-i\;\textsf{stit}]\varphi$ if and only if $S,(b^{}_{i},c^{}_{-i})\models [N-i\;\textsf{stit}]\varphi$, we get

$$\begin{aligned} P^{}_{i}(\varphi \mid a^{}_{i})&= P^{}_{i}(\varphi \wedge \lnot [N-i\;\textsf{stit}]\varphi \mid a^{}_{i}) + P^{}_{i}(\varphi \wedge [N-i\;\textsf{stit}]\varphi \mid a^{}_{i})\\&= P^{}_{i}(\varphi \wedge \lnot [N-i\;\textsf{stit}]\varphi \mid a^{}_{i}) + P^{}_{i}(\varphi \wedge [N-i\;\textsf{stit}]\varphi \mid b^{}_{i}). \end{aligned}$$

So, $$P^{}_{i}(\varphi \mid a^{}_{i}) \ge P^{}_{i}(\varphi \mid b^{}_{i})$$ entails $$P^{}_{i}(\varphi \wedge \lnot [N-i\;\textsf{stit}]\varphi \mid a^{}_{i})\ge P^{}_{i}(\varphi \wedge \lnot [N-i\;\textsf{stit}]\varphi \mid b^{}_{i})$$.

Take an arbitrary $c^{}_{-i}\in A^{}_{-i}$. We prove that $S,(b^{}_{i},c^{}_{-i})\models [i\;\textsf{caus}]\varphi$ entails $S,(b^{}_{i},c^{}_{-i})\models \lnot [N-i\;\textsf{stit}]\varphi$. Suppose $S,(b^{}_{i},c^{}_{-i})\models [i\;\textsf{caus}]\varphi$. We argue by contradiction. Assume $S,(b^{}_{i},c^{}_{-i})\models [N-i\;\textsf{stit}]\varphi$. Given our assumption on *S*, we can conclude that $S,(b^{}_{i},c^{}_{-i})\models [i\;\textsf{stit}]\varphi$. Hence, $P^{}_{i}(\varphi \mid b^{}_{i}) = 1 \le P^{}_{i}(\varphi \mid a^{}_{i})$. The assumption that $P^{}_{i}$ has full support entails that $S,(a^{}_{i},c^{}_{-i})\models [i\;\textsf{stit}]\varphi$. Contradiction.

Hence,

$$\begin{aligned} P^{}_{i}([i\;\textsf{caus}]\varphi \mid b^{}_{i})&= P^{}_{i}(\varphi \wedge \lnot [N-i\;\textsf{stit}]\varphi \wedge [i\;\textsf{caus}]\varphi \mid b^{}_{i}) \\&\le P^{}_{i}(\varphi \wedge \lnot [N-i\;\textsf{stit}]\varphi \mid b^{}_{i}) \\&\le P^{}_{i}(\varphi \wedge \lnot [N-i\;\textsf{stit}]\varphi \mid a^{}_{i}) = P^{}_{i}([i\;\textsf{caus}]\varphi \mid a^{}_{i}). \end{aligned}$$

($\Leftarrow$) Consider any action $a^{}_{-i}\in A^{}_{-i}$ $=A^{}_{j}$. We first consider two basic cases, before dealing with the most complex case. First, assume $S,(a^{}_{i},a^{}_{-i})\models [i\;\textsf{stit}]\varphi$. Then, we immediately get $P^{}_{i}(\varphi \mid a^{}_{i}) = 1 \ge P^{}_{i} (\varphi \mid b^{}_{i})$. Second, assume $S,(a^{}_{i},a^{}_{-i})\models \Box \varphi$. Then, we immediately get $P^{}_{i}(\varphi \mid a^{}_{i}) = 1 = P^{}_{i} (\varphi \mid b^{}_{i})$.

In the remainder of the proof we assume that $S,(a^{}_{i},a^{}_{-i})\models \lnot [i\;\textsf{stit}]\varphi \wedge \Diamond \lnot \varphi$. Notice that this entails that for every $c^{}_{-i}\in A^{}_{-i}$ it holds that $S,(a^{}_{i},c^{}_{-i})\models \lnot [i\;\textsf{stit}]\varphi \wedge \Diamond \lnot \varphi$. Given our assumption on *S*, this entails that for any $c^{}_{-i}\in A^{}_{-i}$ we have $S,(a^{}_{i},c^{}_{-i})\models [i\;\textsf{caus}]\varphi \leftrightarrow [i\;\textsf{butfor}]\varphi$. Hence,

$$\begin{aligned} P^{}_{i}([i\;\textsf{caus}]\varphi \mid a^{}_{i})&= P^{}_{i}(\varphi \wedge \lnot [N-i\;\textsf{stit}]\varphi \mid a^{}_{i}). \end{aligned}$$

Moreover, since for any $c^{}_{-i}\in A^{}_{-i}$ it holds that $S,(a^{}_{i},c^{}_{-i})\models [N-i\;\textsf{stit}]\varphi$ if and only if $S,(b^{}_{i},c^{}_{-i})\models [N-i\;\textsf{stit}]\varphi$, we get

$$\begin{aligned} P^{}_{i}(\varphi \mid a^{}_{i})&= P^{}_{i}(\varphi \wedge \lnot [N-i\;\textsf{stit}]\varphi \mid a^{}_{i}) + P^{}_{i}(\varphi \wedge [N-i\;\textsf{stit}]\varphi \mid a^{}_{i}) \\&= P^{}_{i}(\varphi \wedge \lnot [N-i\;\textsf{stit}]\varphi \mid a^{}_{i}) + P^{}_{i}(\varphi \wedge [N-i\;\textsf{stit}]\varphi \mid b^{}_{i}) \\&= P^{}_{i}([i\;\textsf{caus}]\varphi \mid a^{}_{i}) + P^{}_{i}(\varphi \wedge [N-i\;\textsf{stit}]\varphi \mid b^{}_{i}). \end{aligned}$$

Take an arbitrary $c^{}_{-i}\in A^{}_{-i}$. We prove that $S,(b^{}_{i},c^{}_{-i})\models [i\;\textsf{caus}]\varphi$ entails $S,(b^{}_{i},c^{}_{-i})\models \varphi \wedge \lnot [N-i\;\textsf{stit}]\varphi$. Assume $S,(b^{}_{i},c^{}_{-i})\models [i\;\textsf{caus}]\varphi$. Notice that this entails $S,(b^{}_{i},c^{}_{-i})\models \varphi$. We argue by contradiction. Suppose $S,(b^{}_{i},c^{}_{-i})\models [N-i\;\textsf{stit}]\varphi$. Given our assumption on *S*, this entails that $S,(b^{}_{i},c^{}_{-i})\models [i\;\textsf{stit}]\varphi$. Moreover, since $S,(b^{}_{i},c^{}_{-i})\models \Diamond \lnot \varphi$, we get $S,(b^{}_{i},c^{}_{-i})\models [i\;\textsf{stit}][i\;\textsf{caus}]\varphi$. Hence, $P^{}_{i}([i\;\textsf{caus}]\varphi \mid b^{}_{i}) = 1 \le P^{}_{i}([i\;\textsf{caus}]\varphi \mid a^{}_{i})$. The assumption that $P^{}_{i}$ has full support entails that $S,(a^{}_{i},c^{}_{-i})\models [i\;\textsf{stit}][i\;\textsf{caus}]\varphi$. In particular, we get $S,(a^{}_{i},c^{}_{-i})\models [i\;\textsf{stit}]\varphi$. Contradiction.

Hence,

$$\begin{aligned} P^{}_{i}(\varphi \wedge \lnot [N-i\;\textsf{stit}]\varphi \mid b^{}_{i}) \ge P^{}_{i}([i\;\textsf{caus}]\varphi \mid b^{}_{i}). \end{aligned}$$

In sum,

$$\begin{aligned} P^{}_{i}(\varphi \mid a^{}_{i})&= P^{}_{i}([i\;\textsf{caus}]\varphi \mid a^{}_{i}) + P^{}_{i}(\varphi \wedge [N-i\;\textsf{stit}]\varphi \mid b^{}_{i}) \\&\ge P^{}_{i}([i\;\textsf{caus}]\varphi \mid b^{}_{i}) + P^{}_{i}(\varphi \wedge [N-i\;\textsf{stit}]\varphi \mid b^{}_{i})\\&\ge P^{}_{i}(\varphi \wedge \lnot [N-i\;\textsf{stit}]\varphi \mid b^{}_{i}) + P^{}_{i}(\varphi \wedge [N-i\;\textsf{stit}]\varphi \mid b^{}_{i})\\&= P^{}_{i}(\varphi \mid b^{}_{i}). \end{aligned}$$

$$\square$$

#### Corollary 5

*Let* *R* *be a responsibility game under risk and let* *i* *in* *N* *be an agent. Suppose that agent* *i*’*s probability distribution has full support and satisfies probabilistic independence. Assume that agent* *i* *satisfies simple causation for* $0$. *Then*:

$$\begin{aligned} \begin{array}{ll} R\models \lnot [i\;\textsf{prom}]1\leftrightarrow \lnot [i\;\textsf{prom}]\lnot [i\;\textsf{caus}]0\\ R \models [i\;\textsf{prom}]0\leftrightarrow [i\;\textsf{prom}][i\;\textsf{caus}]0. \\ \end{array} \end{aligned}$$

*Assume that agent* *i* *satisfies simple causation for* $1$. *Then*:

$$\begin{aligned} \begin{array}{ll} R \models \lnot [i\;\textsf{prom}]1\leftrightarrow \lnot [i\;\textsf{prom}][i\;\textsf{caus}]1\\ R \models [i\;\textsf{prom}]0\leftrightarrow [i\;\textsf{prom}]\lnot [i\;\textsf{caus}]1. \\ \end{array} \end{aligned}$$

#### Proof

I prove the first two validities, since the last two validities can be proved in a similar way. The second validity follow immediately from Theorem 2 under the assumption that $R\models [i\;\textsf{caus}]\varphi \leftrightarrow ([i\;\textsf{dstit}]\varphi \vee [i\;\textsf{butfor}]\varphi )$ for $\varphi \equiv 0$. The first item follows from the following observation. Take any $b^{}_{i}$ in $A^{}_{i}$. Then, the following are equivalent:

1. $$P^{}_{i}(1\mid b^{}_{i}) > P^{}_{i}(1\mid a^{}_{i})$$;
2. $$P^{}_{i}(0\mid b^{}_{i}) < P^{}_{i}(0\mid a^{}_{i})$$;
3. $$P^{}_{i}([i\;\textsf{caus}]0\mid b^{}_{i}) < P^{}_{i}([i\;\textsf{caus}]0\mid a^{}_{i})$$; and
4. $$P^{}_{i}(\lnot [i\;\textsf{caus}]0\mid b^{}_{i}) > P^{}_{i}(\lnot [i\;\textsf{caus}]0\mid a^{}_{i})$$.

Notice that the equivalence of (2) and (3) only relies on the assumption that $R\models [i\;\textsf{caus}]\varphi \leftrightarrow ([i\;\textsf{dstit}]\varphi \vee [i\;\textsf{butfor}]\varphi )$ for $\varphi \equiv 0$, as opposed to for $\varphi \equiv 1$. $\square$

### Responsibility Games Under Uncertainty

Before proceeding with the proofs, let me introduce some notation. Consider any responsibility game $S=\langle N, (A^{}_{i}), V\rangle$. For any formula $\varphi$, any agent *i*, and any individual action $a^{}_{i}$, we use $(\varphi \mid a^{}_i)$ to denote $\{b^{}_{-i}\in A^{}_{-i}\mid S,(a^{}_{i}, b^{}_{-i})\models \varphi \}$. It is easy to see that, for any individual actions $a^{}_{i}$ and $b^{}_{i}$ it holds that $a^{}_{i}\succeq _{\varphi } b^{}_{i}$ if and only if $(\varphi \mid a^{}_{i})\supseteq (\varphi \mid b^{}_{i})$.

#### Observation 1

*Let* *R* *be a responsibility game under risk, let* $i\in N$ *be an agent, and let* $\varphi$ *be a formula*. *Let* $a^{}_i$ *and* $b^{}_i$ *be individual actions available to agent* *i*. *Assume that the probability distribution* $P^{}_{i}$ *satisfies probabilistic independence and has full support. Then*:

- *if there is no* $b^{}_{i}$ *in* $A^{}_{i}$ *such that* $P^{}_i(\varphi \mid b^{}_i) > P^{}_i(\varphi \mid a^{}_i)$, *then* $a^{}_{i}$ *is admissible with respect to* $\varphi$.

#### Proof

Notice that $a^{}_i \succ _{\varphi } b^{}_i$ entails $P^{}_i(\varphi \mid a^{}_i) > P^{}_i(\varphi \mid b^{}_i)$. After all, the assumptions together imply that $(\varphi \mid a^{}_{i})\supset (\varphi \mid b^{}_{i})$ entails that $P^{}_i(\varphi \mid a^{}_i) > P^{}_i(\varphi \mid b^{}_i)$.

We argue by contradiction to prove the desired statement. Suppose $a^{}_{i}$ is not admissible with respect to $\varphi$. Let $c^{}_{i}$ in $A^{}_{i}$ be such that $c^{}_{i}\succ _{\varphi } a^{}_{i}$. Hence, we get $P^{}_i(\varphi \mid c^{}_i) > P^{}_i(\varphi \mid a^{}_i)$, which contradicts the antecedent. $\square$

#### Theorem 1

*Let* *S* *be a responsibility game*, *let* $i\in N$ *be an agent, and let* $\varphi$ *be a formula*. *Let* $a^{}_i$ *and* $b^{}_i$ *be individual actions available to agent* *i*. *Then*:

$$\begin{aligned} \begin{array}{lll} a^{}_i \succeq _{\varphi } b^{}_i &{} {iff} &{} a^{}_i \succeq _{[i\;\textsf{caus}]\varphi } b^{}_i. \\ \end{array} \end{aligned}$$

*As a consequence*, $a^{}_{i}$ *is admissible with respect to* $\varphi$ *if and only if* $a^{}_{i}$ *is admissible with respect to* $[i\;\textsf{caus}]\varphi$. *That is*:

$$\begin{aligned} \begin{array}{lll} \text {Admissible}_{S}(i,\varphi ) &{} = &{} \text {Admissible}_{S}(i,[i\;\textsf{caus}]\varphi ). \\ \end{array} \end{aligned}$$

#### Proof

We need to show that

$$\begin{aligned} \begin{array}{lll} (\varphi \mid a^{}_{i})\supseteq (\varphi \mid b^{}_{i}) &{} iff &{} ([i\;\textsf{caus}]\varphi \mid a^{}_{i})\supseteq ([i\;\textsf{caus}]\varphi \mid b^{}_{i}). \\ \end{array} \end{aligned}$$

($\Rightarrow$) Suppose $a^{}_{i}\succeq _{\varphi } b^{}_i$. Take an arbitrary $c^{}_{-i}\in A^{}_{-i}$. Suppose $(b^{}_{i}, c^{}_{-i})\models [i\;\textsf{caus}]\varphi$. Then there is a ${\mathcal {G}}\subseteq N$ such that $i\in {\mathcal {G}}$ and $(b^{}_{i}, c^{}_{-i})\models [{\mathcal {G}}\;\textsf{stit}]\varphi \wedge \lnot [{\mathcal {G}}-i\;\textsf{stit}]\varphi$. Take an arbitrary $d^{}_{-{\mathcal {G}}}\in A^{}_{-{\mathcal {G}}}$. Let $a'_{{\mathcal {G}}}:=(a^{}_{i}, c^{}_{{\mathcal {G}}-i})$ and $b'_{{\mathcal {G}}}:= (b^{}_{i}, c^{}_{{\mathcal {G}}-i})$. Since $a^{}_{i}\succeq _{\varphi } b^{}_i$ and $(b^{}_{i}, c^{}_{-i})\models [{\mathcal {G}}\;\textsf{stit}]\varphi$, we get $(b'_{{\mathcal {G}}},d^{}_{-{\mathcal {G}}})\models \varphi$ and, therefore, $(a'_{{\mathcal {G}}},d^{}_{-{\mathcal {G}}})\models \varphi$. Since $d^{}_{{\mathcal {G}}}\in A^{}_{{\mathcal {G}}}$ was arbitrary, we have $(a^{}_{i}, c^{}_{-i})\models [{\mathcal {G}}\;\textsf{stit}]\varphi$. Moreover, since $(a^{}_{i}, c^{}_{-i})^{}_{{\mathcal {G}}-i}=(b^{}_{i}, c^{}_{-i})^{}_{{\mathcal {G}}-i}$, we get $(a^{}_{i}, c^{}_{-i})\models \lnot [{\mathcal {G}}-i\;\textsf{stit}]\varphi$. Hence, $(a^{}_{i}, c^{}_{-i})\models [i\;\textsf{caus}]\varphi$, as desired. Since $c^{}_{-i}\in A^{}_{-i}$ was arbitrary, we get $a^{}_{i}\succeq _{[i\;\textsf{caus}]\varphi } b^{}_i$.

($\Leftarrow$) Suppose $a^{}_{i}\succeq _{[i\;\textsf{caus}]\varphi } b^{}_i$. Take an arbitrary $c^{}_{-i}\in A^{}_{-i}$. Suppose $(b^{}_{i}, c^{}_{-i})\models \varphi$. We separate two cases. Case (i): assume $(b^{}_{i}, c^{}_{-i})\models [i\;\textsf{caus}]\varphi$. Then, by assumption, we get $(a^{}_{i}, c^{}_{-i})\models [i\;\textsf{caus}]\varphi$ and thus $(a^{}_{i}, c^{}_{-i})\models \varphi$, as desired. Case (ii): assume $(b^{}_{i}, c^{}_{-i})\models \lnot [i\;\textsf{caus}]\varphi$. Since the games under consideration are deterministic, we have $(b^{}_{i}, c^{}_{-i})\models [N\;\textsf{stit}]\varphi$. Since agent *i* does not causally contribute to bringing about $\varphi$, we must have $(b^{}_{i}, c^{}_{-i})\models [N-i\;\textsf{stit}]\varphi$. Since $(a^{}_{i}, c^{}_{-i})^{}_{N-i}=(b^{}_{i}, c^{}_{-i})^{}_{N-i}$, we get $(a^{}_{i}, c^{}_{-i})\models [N-i\;\textsf{stit}]\varphi$ and therefore $(a^{}_{i}, c^{}_{-i})\models \varphi$, as desired. Since $c^{}_{-i}\in A^{}_{-i}$ was arbitrary, we get $a^{}_{i}\succeq _{\varphi } b^{}_i$. $\square$

#### Observation 2

*Let* *S* *be a responsibility game, let* $i\in N$ *be an agent, and let* $\varphi$ *be a formula. Let* $a^{}_i$ and $b^{}_i$ *be individual actions available to agent* *i*. *Then*:

$$\begin{aligned} \begin{array}{lll} a^{}_i \succeq _{\varphi } b^{}_i &{} iff &{} b^{}_i \succeq _{\lnot \varphi } a^{}_i. \\ \end{array} \end{aligned}$$

#### Proof

By definition, the following are equivalent: (1) $a^{}_{i}\succeq _{\varphi } b^{}_{i}$; (2) $(\varphi \mid a^{}_{i}) \supseteq (\varphi \mid b^{}_{i})$; (3) $(\lnot \varphi \mid b^{}_{i}) \supseteq (\lnot \varphi \mid a^{}_{i})$; (4) $b^{}_{i}\succeq _{\lnot \varphi } a^{}_{i}$. $\square$

#### Observation 3

*Let* *R* *be a responsibility game under risk, let* $i\in N$ *be an agent, and let* $\varphi$ *be a formula. Suppose that agent* *i*’s *probability distribution has full support and satisfies probabilistic independence. Assume that agent* *i**’s actions are totally ordered for* $\varphi$. Let $a^{}_{i}$ *and* $b^{}_{i}$ *be individual actions available to agent* *i*. *Then*:

$$\begin{aligned} \begin{array}{lll} a^{}_i \succeq _{\varphi } b^{}_i &{} iff &{} P^{}_{i}(\varphi \mid a^{}_i) \ge P^{}_{i}(\varphi \mid b^{}_i). \\ \end{array} \end{aligned}$$

*Consequently, any action is admissible in the responsibility game* *S* *if and only if that action promotes the probability in the responsibility game under risk* *R*.

#### Proof

($$\Rightarrow$$) Trivial.

($\Leftarrow$) Assume $P^{}_{i}(\varphi \mid a^{}_i) \ge P^{}_{i}(\varphi \mid b^{}_i)$. We argue by contradiction. Suppose $a^{}_{i}\nsucceq _{\varphi } b^{}_{i}$. Since $\succeq _{\varphi }$ is a total ordering on $A^{}_{i}$, we get $b^{}_{i}\succeq _{\varphi } a^{}_{i}$. Hence, $(\varphi \mid b^{}_{i})\supset (\varphi \mid a^{}_{i})$. Since $P^{}_{i}$ has full support, this entails $P^{}_{i}(\varphi \mid a^{}_i) < P^{}_{i}(\varphi \mid b^{}_i)$. Contradiction. $\square$

## Footnotes

1 Note that moral responsibility for outcomes is different from mere causal responsibility and from prospective moral responsibility (i.e. moral duties or obligations).

2 The idea that the ability to do otherwise plays a key role in responsibility attributions goes back to Aristotle (see *Nichomachaen Ethics*, 2019; 1113b7-8) and it is closely related to the principle of alternative possibilities (Frankfurt, 1969; Van Inwagen, 1999). More precisely, my formalization is related to recent work on moral responsibility and the avoidance of blame (McKenna, 1997; Wyma, 1997; Otsuka, 1998; Hetherington, 2003). Formalisms that capture this idea in a somewhat different way than mine are presented by Vallentyne (2008) and Braham and van Hees (2012).

3 I will largely eschew the structural-equations models of causation that have become influential in the literature on causation.

4 In line with standard terminology in decision theory, see Luce and Raiffa (1957). In contrast, see (Hansson, 2013) for a critical discussion of this usage and other connotations of risk.

5 It will become clear that the semantics I propose are naturally related to the analysis of obligations by Horty (2001) and the analysis of responsibility in Tamminga and Duijf (2017), Duijf and Van De Putte (2022) and Duijf (2022).

6 Also see the three conditions for fitness to be held responsible in Pettit (2007).

7 The constant $0$ is related to the “violation” constant in the literature on deontic logic, which is meant to express that some (relevant) normative requirement is violated. Such a constant is central in the Anderson-Kanger reduction of deontic logic to modal logic.

8 One can view this language as being restricted to a single morally relevant propositional variable $0$, which represents a particular morally undesirable state of affairs. In future work, one could generalize this framework to encompass a set of propositional variables that are morally relevant (also see Sect. 9).

9 A reviewer suggested that the morally undesirable state of affairs may be context-dependent: for instance, a death may be morally bad in a situation where the person is killed (without any moral justification) and it may not be morally bad in a situation where the person dies of natural causes. My framework is compatible with this idea in the sense that the constant 0 denotes a particular morally undesirable state of affairs, regardless of whether this state of affairs is considered to be universally morally bad or only morally bad in a given context.

10 I do not consider the criterion formalized by $\lnot [i\;\textsf{caus}]\lnot 0$ ($\equiv \lnot [i\;\textsf{caus}]1$), which characterizes the fact that agent *i* did not causally contribute to realizing $1$. Since I’m interested in studying moral responsibility for actual outcomes, the condition formalized by $\lnot [i\;\textsf{caus}]1$ will always be satisfied. After all, it seems plausible to assume that the formula $[i\;\textsf{caus}]\varphi \rightarrow \varphi$ is valid. Or, equivalently, $\lnot \varphi \rightarrow \lnot [i\;\textsf{caus}]\varphi$ is valid. In particular, $0\rightarrow \lnot [i\;\textsf{caus}]1$ is valid. In fact, the semantics discussed in the second part of the paper will validate this formula. In any case, notice that, under this assumption, we get that $[i\;\textsf{caus}]0\rightarrow \lnot [i\;\textsf{caus}]1$ is valid. This means that there is a *logical* relation between these two criteria.

11 Let me add two remarks on criteria (2) and (3). It is natural to think that these two notions of moral responsibility relate to the idea of choosing the *best* course of action and choosing a *satisfactory* course of action, respectively. For instance, criterion (2) $[i\;\textsf{prom}]0$ says that one can avoid responsibility by simply not choosing the worst course of action. That is, any course of action that is better than the worst one is satisfactory. Alternatively, it is plausible to think that moral responsibility requires that one performs an action that is better than some default course of action or one that exceeds some reference point. Criteria (2) and (3) would then relate to a *minimal* or a *maximal* reference point. There may be plausible *intermediate* versions of this threshold idea and it might be interesting to use the present framework to study such intermediate versions in future work.

12 Although Braham and van Hees (2012, 2018) call this the avoidance condition, it should not be confused with my negative condition of an alternative possibility. They model causal contribution using the NESS test; which is also our approach in Sect. 4.

13 From a purely syntactical perspective, one may ask why we have not considered notions of moral responsibility that result from swapping the $[i\;\textsf{prom}]$ operator with the $[i\;\textsf{caus}]$ operator in the four notions we mentioned last. It seems natural to think that whether or not a given agent promotes the realization of a given state of affairs solely depends on that agent. If this is correct, then it is plausible to think that $[i\;\textsf{caus}][i\;\textsf{prom}]\varphi$ is logically equivalent to $[i\;\textsf{prom}]\varphi$. In fact, my formalism validates this intuition: this logical equivalence obtains both in scenarios of decision-making under risk and under uncertainty.

14 Game-theoretical models have a long history in philosophy; these particular responsibility games are very similar to the ones used by Braham and van Hees (2012, 2018) and to deontic game models (Tamminga and Hindriks, 2020).

15 The representation theorems by Ramsey (1931), von Neumann and Morgenstern (1944), and Savage (1954) lay the foundations for the so-called revealed preference interpretation of utilities and probabilities. For a helpful exposition of the interpretation of the models in rational choice theory, see Okasha (2016). A decision theorist can view my formal analysis as being restricted to only binary values or utilities. In future work, one might consider generalizing responsibility games to include arbitrary real-valued utilities.

16 Seminal works include contributions by Chellas, von Kutschera, Kanger, Pörn, Segerberg, and von Wright. See Belnap (1991) and Segerberg (1992) for a more extensive historical overview and appropriate references.

17 In the tradition of stit theory, this operator is known as the Chellas stit operator. The literature also includes other stit operators, most notably the achievement stit, the deliberative stit, and the strategic stit operators (see, for instance, (Horty, 2001)).

18 It may be helpful to note some logical properties of these operators. The $[{\mathcal {G}}\;\textsf{stit}]$ and $\Box$ operator satisfy the S5 properties. Moreover, the *independence of agency* principle is also valid (see (Horty, 2001, § 2.4) for a useful discussion), which is formalized by the schema $\bigwedge _{i\in N}\Diamond [i\;\textsf{stit}]\varphi _i \rightarrow \Diamond (\bigwedge _{i\in N}[i\;\textsf{stit}]\varphi _i)$ which says that each agent is able to perform any of her available actions, regardless of the actions performed by others.

19 It may be helpful to note that stit theory is related to $\alpha$-effectivity. Agent *i* is $\alpha$-effective for a given state of affairs *X* if and only if there is an action $a^{}_{i}\in A^{}_{i}$ such that for all $a^{}_{-i}\in A^{}_{-i}$ it holds that $(a^{}_{i}, a'_{-i})\in X$. Then, agent *i* is $\alpha$-effective for a state of affairs *X* if and only if it is possible that agent *i* sees to it that *X* obtains. The notion of $\alpha$-effectivity was first defined by Moulin and Peleg (1982) and is central to Pauly’s (2002) characterization of strategic games in terms of effectivity functions.

20 This view is related to the notion of “responsibility by control” in (Fleurbaey, 1995, p. 684): “Responsibility by control is assigned to an agent on a particular variable when this agent has full control over the value of this variable.”

21 Note that the second conjunct is equivalent to $\langle N-i\;\textsf{stit}\rangle \lnot \varphi$.

22 The popular approach to causal modelling based on structural-equations models will be largely eschewed here (Chockler and Halpern, 2004; Halpern and Pearl, 2005; Woodward and Hitchcock, 2003). The current logical framework seems to be straightforwardly applicable to these causal models. Nevertheless, developing this framework in more detail is a significant research project that has to be left to another occasion.

23 As the name suggests, there is an alternative notion of a *strong* NESS condition. A strong NESS condition strengthens the second requirement in the definition below to the requirement that (2’) for all $j\in {\mathcal {G}}$ it holds that $S,a\models \lnot [{\mathcal {G}}-j\;\textsf{stit}]\varphi$. For reasons of scope, I leave the study of this alternative semantics to another occasion.

24 Regarding the basic logical properties of the $[i\;\textsf{caus}]$ operator, it may be helpful to note that it is non-monotonic (and, hence yields a non-normal modal logic). In particular, the operator validates an anti-necessitation rule ($\models \varphi$ entails $\models \lnot [i\;\textsf{caus}]\varphi$) and it falsifies the K axiom. Moreover, it is easy to see that the T axiom is valid: $\models [i\;\textsf{caus}]\varphi \rightarrow \varphi$.

25 For example, Lorini et al. (2014, p. 1324) model causal responsibility as “either the agent sees to it that $\varphi$ is true or she could have prevented $\varphi$ from being true”, which is represented by the formula: $[i\;\textsf{dstit}]\varphi \vee \langle -i \;\textsf{stit}\rangle \lnot [N\;\textsf{stit}]\varphi$. In responsibility games, the NESS test is more permissive for actual outcomes: it is easy to verify that $\models \varphi \rightarrow \big ( ([i\;\textsf{dstit}]\varphi \vee \langle -i\;\textsf{stit}\rangle \lnot [N\;\textsf{stit}]\varphi )\rightarrow [i\;\textsf{caus}]\varphi \big )$.

26 More explicitly, and ignoring the differences between Chellas stit and achievement stit, agent *i* is a weak NESS condition for $\varphi$ if and only if agent *i* is “not a mere bystander for $\varphi$” (Belnap and Perloff, 1993, p. 40—notation adapted). Moreover, agent *i* “is essential for $[{\mathcal {G}}\;\textsf{stit}]\varphi$” (Belnap and Perloff, 1993, p. 40—notation adapted) if and only if items 1. and 2. of Definition 5 are satisfied.

27 In contrast, Hansson (2013, p. 8) argues that “the identification of risk with probability has the problematic feature of making risk insensitive to the severity of the undesired outcome”.

28 Accordingly, the subjective interpretation of probabilities aligns with an often discussed epistemic condition for moral responsibility: “The ‘epistemic condition’ corresponds to the excuse of ignorance. It captures the intuition that an agent is responsible only if he both knows the particular facts surrounding his action, and acts with the proper sort of beliefs and intentions” (Fischer and Ravizza, 1998, p. 13).

29 Similarly, Zimmerman (2008, pp. 2, 5, and 19) distinguishes between objective and subjective views: “The Objective View (first formulation): An agent ought to perform an act if and only if it is the best option that he (or she) has. ...The Subjective View (first and only formulation): An agent ought to perform an act if and only if he believes that it is the best option that he has”.

30 This subjective interpretation is in line with an epistemic condition for moral responsibility and is distinct from probabilistic approaches to causation (which normally rely on an objective interpretation of the probabilities) (Vallentyne, 2008). For reasons of scope, the logical study of probabilistic approaches to causation has to be left to another occasion.

31 It may be helpful to indicate a notational convention: we use *S*, $S^{}_{1}$, $S'$, etc. to denote a responsibility game and we will use *R*, $R^{}_{1}$, $R'$, etc. to denote a responsibility game under risk.

32 Regarding the basic logical properties of the $[i\;\textsf{prom}]$ operator, it may be helpful to note that it yields a non-normal modal logic. In particular, the operator validates the necessitation rule, but falsifies the K axiom.

33 In particular, remark that for any two actions in $A^{}_{i}$, one is at least as good as the other. Moreover, there is an action $a^{}_{i}$ in $A^{}_{i}$ that is at least as good as any other action in $A^{}_{i}$. Things are different for the ordering on individual actions in decision-making under uncertainty presented in Sect. 7.

34 It should be noted that Braham and van Hees (2012) add the caveat that an agent is morally responsible if her action is not *eligible*. Regrettably, they leave the concept of eligibility unanalysed and, instead, they give it formal content by adding an eligibility parameter to their models.

35 It follows from the fact that the $[i\;\textsf{stit}]$ satisfies the S5 principles that the following formulas are also valid: $[i\;\textsf{stit}]\varphi \rightarrow [i\;\textsf{prom}][i\;\textsf{stit}]\varphi$ and $[i\;\textsf{stit}]\varphi \rightarrow [i\;\textsf{stit}][i\;\textsf{prom}]\varphi$.

36 The first validity can be verified by considering two cases depending on whether $\Box \varphi$ holds; the second invalidity can be observed in a responsibility game under risk where $\Box \varphi$ holds; and the third invalidity can be demonstrated using a responsibility game under risk where agent *i*’s probability distribution does not have full support.

37 This follows from Corollaries 1 and 2 (see below) and Observation 5 (see the appendix).

38 Corollary 1 follows from Observation 4, which is stated and proved in the appendix.

39 An example where the second condition holds would be responsibility game $R_4$. Let me give an example where the second condition of Corollary 1 fails: consider the responsibility game under risk $R'_{4}$ that extends $S_{4}$ with the conditional probability distribution $P^{}_i$ that assigns probability one to $c^{}_{j}$, regardless of agent *i*’s action.

40 Corollary 2 follows from Observation 4, which is stated and proved in the appendix.

41 Notice that $[i\;\textsf{caus}]0$ is not true at any of the action profiles that validate $1$. Moreover, observe that $[i\;\textsf{caus}]0$ is true at each of $(b^{}_{i},a^{}_{j}, a^{}_{k})$, $(a^{}_{i},b^{}_{j}, a^{}_{k})$, and $(a^{}_{i},a^{}_{j}, b^{}_{k})$ since agent *i* is a but-for condition for $0$ at those profiles. By considering the group $\{i,k\}$, one can observe that $[i\;\textsf{caus}]0$ also holds at $(a^{}_{i},b^{}_{j},b^{}_{k})$. In contrast, one can easily verify that $[i\;\textsf{caus}]0$ does not hold at $(b^{}_{i},b^{}_{j}, b^{}_{k})$.

42 It is easy to verify that the decision-theoretic notion of admissibility presented below satisfies both of these requirements.

43 Savage (1954, p. 21) writes: “I know of no other extralogical principle governing decisions that finds such ready acceptance.” My personal inspiration is from Horty (1996, 2001), who provided a similar analysis in deontic logic, which is the formal study of obligations and permissions, by introducing “an ordering on actions available to the agent through a state-by-state comparison of their results”, where “we will identify the states confronting the agent at any given moment with the possible patterns of actions that might be performed at that moment by all other agents” (Horty, 2001, p. 67 and p. 66).

44 Horty in fact uses “weakly dominates” for what I call “simply dominates”, and “strongly dominates” for what I call “weakly dominates”. My choice of terminology follows common practice in decision and game theory.

45 Weak dominance is distinct from strict dominance. Given a utility function *u*, an action $a^{}_{i}$ strictly dominates $b^{}_{i}$ if and only if for all $c^{}_{-i}\in A^{}_{-i}$ we have $u(a^{}_{i},c^{}_{-i})> u^{}_\varphi (b^{}_{i},c^{}_{-i})$. Avoiding inadmissible actions is thus more restrictive than avoiding *strictly* dominated actions. Note that I do not employ iterated admissibility, i.e. iterated deletion of dominated actions (see the discussion in Kohlberg and Mertens (1986, Section 2.7) and the discussion of epistemic characterizations of iterated admissibility in Brandenburger et al. (2008, Section 2.6). One of the main reasons for refraining from doing so is that iterated admissibility is subject to some paradoxes: for instance, it is well known that the order in which dominated strategies are eliminated can affect the outcome of the process.

46 It is easy to verify that each of these assumptions is necessary for the validity of this statement. In particular, it is easy to construct a responsibility game under risk that falsifies this statement, where the scenario includes probability distributions that violate probabilistic independence. The same holds for the assumption that the probability distributions have full support.

47 Also see Jeffrey (1965) and Gibbard and Harper (1978).

48 To be precise, ${\mathcal {C}}\models \chi$ holds if and only if for every responsibility game under risk *R* in ${\mathcal {C}}$ and any action profile *a* it holds that $R,a\models \chi$.

49 For example, Braham and van Hees (2012, § 7.3) endorse one of the complex criteria for moral responsibility and discuss a two-player version of a Frankfurt’s case that involves probability distributions that lack full support to argue that an agent can be morally responsible for a certain outcome even though that agent has no influence on the probability that the outcome is realized.
